# Multiparametric detection and outcome prediction of pancreatic cancer involving dual-energy CT, diffusion-weighted MRI, and radiomics

**DOI:** 10.1186/s40644-023-00549-8

**Published:** 2023-04-18

**Authors:** Vitali Koch, Nils Weitzer, Daniel Pinto Dos Santos, Leon D. Gruenewald, Scherwin Mahmoudi, Simon S. Martin, Katrin Eichler, Simon Bernatz, Tatjana Gruber-Rouh, Christian Booz, Renate M. Hammerstingl, Teodora Biciusca, Nicolas Rosbach, Aynur Gökduman, Tommaso D’Angelo, Fabian Finkelmeier, Ibrahim Yel, Leona S. Alizadeh, Christof M. Sommer, Duygu Cengiz, Thomas J. Vogl, Moritz H. Albrecht

**Affiliations:** 1 Department of Diagnostic and Interventional Radiology, University Hospital Frankfurt, Theodor-Stern-Kai 7, Frankfurt am Main, 60590 Germany; 2 Department of Biomedical Sciences and Morphological and Functional Imaging, University Hospital Messina, Messina, Italy; 3 Department of Internal Medicine, University Hospital Frankfurt, Frankfurt Am Main, Germany; 4 Clinic of Diagnostic and Interventional Radiology, Heidelberg University Hospital, Heidelberg, Germany; 5 Department of Radiology, University of Koc School of Medicine, Istanbul, Turkey

**Keywords:** Multidetector computed tomography, Pancreatic cancer, Survival, Diffusion magnetic resonance imaging, Dual-energy computed tomography

## Abstract

**Background:**

The advent of next-generation computed tomography (CT)- and magnetic resonance imaging (MRI) opened many new perspectives in the evaluation of tumor characteristics. An increasing body of evidence suggests the incorporation of quantitative imaging biomarkers into clinical decision-making to provide mineable tissue information. The present study sought to evaluate the diagnostic and predictive value of a multiparametric approach involving radiomics texture analysis, dual-energy CT-derived iodine concentration (DECT-IC), and diffusion-weighted MRI (DWI) in participants with histologically proven pancreatic cancer.

**Methods:**

In this study, a total of 143 participants (63 years ± 13, 48 females) who underwent third-generation dual-source DECT and DWI between November 2014 and October 2022 were included. Among these, 83 received a final diagnosis of pancreatic cancer, 20 had pancreatitis, and 40 had no evidence of pancreatic pathologies. Data comparisons were performed using chi-square statistic tests, one-way ANOVA, or two-tailed Student’s t-test. For the assessment of the association of texture features with overall survival, receiver operating characteristics analysis and Cox regression tests were used.

**Results:**

Malignant pancreatic tissue differed significantly from normal or inflamed tissue regarding radiomics features (overall *P* < .001, respectively) and iodine uptake (overall *P* < .001, respectively). The performance for the distinction of malignant from normal or inflamed pancreatic tissue ranged between an AUC of ≥ 0.995 (95% CI, 0.955–1.0; *P* < .001) for radiomics features, ≥ 0.852 (95% CI, 0.767–0.914; *P* < .001) for DECT-IC, and ≥ 0.690 (95% CI, 0.587–0.780; *P* = .01) for DWI, respectively. During a follow-up of 14 ± 12 months (range, 10–44 months), the multiparametric approach showed a moderate prognostic power to predict all-cause mortality (c-index = 0.778 [95% CI, 0.697–0.864], *P* = .01).

**Conclusions:**

Our reported multiparametric approach allowed for accurate discrimination of pancreatic cancer and revealed great potential to provide independent prognostic information on all-cause mortality.

**Supplementary Information:**

The online version contains supplementary material available at 10.1186/s40644-023-00549-8.

## Background

Despite new diagnostic and therapeutic approaches aiming at improving survival, the prognosis of patients with pancreatic cancer remains poor with a dismal 5-year survival rate of less than 5% [1]. Even after surgical resection, pancreatic cancer tends to reoccur with a 5-year survival of only 25% in surgically treated patients [2]. Therefore, early detection of pancreatic cancer seems of tremendous importance to avoid poor outcomes. Considering their frequently subtle imaging manifestation due to small and isoattenuating appearance, early parenchymal changes are often misdiagnosed or even missed in computed tomography (CT) or magnetic resonance imaging (MRI) [3].

Several studies have shown that long-term survival is affected by a multitude of different factors including tumor size, presence of metastasis, heterogeneity, or histologic differentiation of malignant tissue [4–7]. Recently published studies revealed strong correlations of intratumoral heterogeneity in malignant pancreatic lesions with tumor recurrence and survival [8–10]. Tumor masses can be quantified non-invasively by using dual-energy computed tomography iodine concentration (DECT-IC) [11, 12] or diffusion-weighted magnetic resonance imaging (DWI) based on the random Brownian motion of water molecules within tissues [13]. Radiomics is a quantitative approach that poses the potential to extract a myriad of texture features from medical imaging by using miscellaneous mathematical extraction algorithms. This rapidly growing discipline aims to establish imaging biomarkers that can assist in risk stratification and outcome prediction.

Given the value of radiomics to add essential information to CT and MRI [13–17], we hypothesized that a multiparametric approach involving all three techniques might be superior to every single diagnostic tool in distinguishing malignant from normal or inflamed parenchyma and in predicting outcomes.

## Methods

The present study was approved by the local institutional review board. Informed consent was obtained from all enrolled participants following the Declaration of Helsinki.

### Study population

**Fig. 1:**
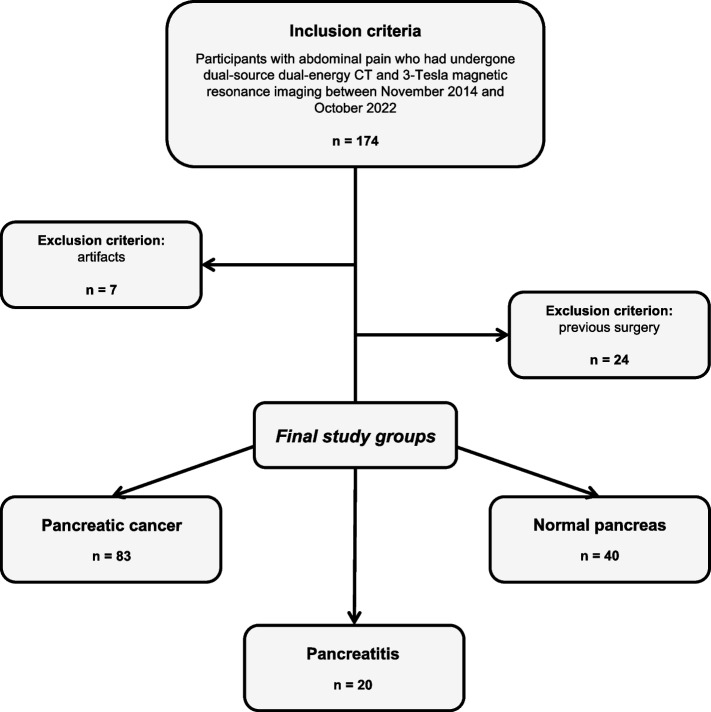
Flowchart of patient inclusion

A total of 174 participants with unspecific abdominal pain who had undergone clinically indicated third-generation dual-source DECT and 3-Tesla MRI at the University Hospital Frankfurt (Frankfurt am Main, Hesse, Germany) between November 2014 and October 2022 were initially considered for study inclusion. To limit possible distortion of statistics between DECT and MRI, we included only data from patients with an examination interval of up to 14 days between the two examinations. Indications to perform an additional MRI scan to DECT included 1) the need for a better characterization of tumor tissue, e. g. the infiltration into adjacent structures, 2) short-term follow-up with concerns about ionizing radiation for another CT imaging, and 3) suboptimal CT findings with a high clinical suspicion of pancreatic cancer. Exclusion criteria were artifacts (*n* = 7) and previous local surgery (*n* = 24). The final study cohort consisted of 143 patients, of whom 83 received a final diagnosis of histologically proven pancreatic cancer (12 of 83 patients were prospectively enrolled for data validation purposes). Another 40 participants had normal pancreatic parenchyma and served as the comparative group, including individuals with gastritis (*n* = 16), gastroesophageal reflux disease (*n* = 11), constipation (*n* = 6), gastroenteritis (*n* = 5), or inflammatory bowel disease (*n* = 2). 20 participants were finally diagnosed with pancreatitis. For retrospective analysis, suitable patients were identified in the picture archiving and communication system (Centricity 4.2; GE Healthcare, Chicago, USA) by searching for the following terms: ‘pancreatic cancer’, ‘pancreatitis’, ‘normal pancreatic parenchyma’, ‘pancreatic adenocarcinoma’, and ‘pancreatic neuroendocrine tumor’. Exclusion criteria were previous tumor resection, imaging artifacts, stent material, or pancreatolithiasis. Figure 1 illustrates the selection process of participants in this study.

### Standard of reference

The final adjudicated diagnosis was based on the histological confirmation in all cases of pancreatic cancer, as well as on clinical and imaging findings at discharge. All data were extracted from medical reports and multidisciplinary tumor board meetings. Tumors were graded according to the 8^th^ edition of TNM classification (clinical and pathological) [18]. The primary endpoint was all-cause mortality. Follow-up was performed by thoroughly checking outcome information including medical reports and external files from referring hospitals. Patient outcome was also collected by phone interviews and questionnaires.

### Dual-energy CT protocol

All CT examinations were performed on a third-generation dual-source DECT scanner equipped with a Somatom Force unit (Siemens Healthineers, Forchheim, Bavaria, Germany). The examination parameters were as follows: tube A, 90 kVp and 220 mAs; tube B, Sn150 kVp [0.64 mm tin filter] and 138 mAs; collimation of 2 × 192 × 0.6 mm; rotation time of 0.5 s; pitch of 0.6. Real-time automatic tube current modulation was applied (CARE Dose 4D; Siemens Healthineers). Images were reconstructed with a slice thickness of 2 mm in 2 mm intervals using advanced modeled iterative reconstruction (ADMIRE, Siemens Healthineers) with a medium smooth reconstruction kernel (Br40). A nonionic intravenous contrast medium was administered (Imeron 400, Bracco, Milan, Italy) at a flow rate of 3 mL/second (dose of 1.2 mL/kg body weight).

All examinations consisted of a standardized triphasic pancreatic mass protocol, including a noncontrast, pancreatic arterial (PAP), and portal venous phase (PVP). Applying bolus triggering, PAP was acquired with a 5-s delay after aortic threshold enhancement of 100 HU. Given greater contrast ratios between tumor and adjacent parenchyma, PAP scans were used consistently for texture analysis.

### Image segmentation and analysis

**Fig. 2:**
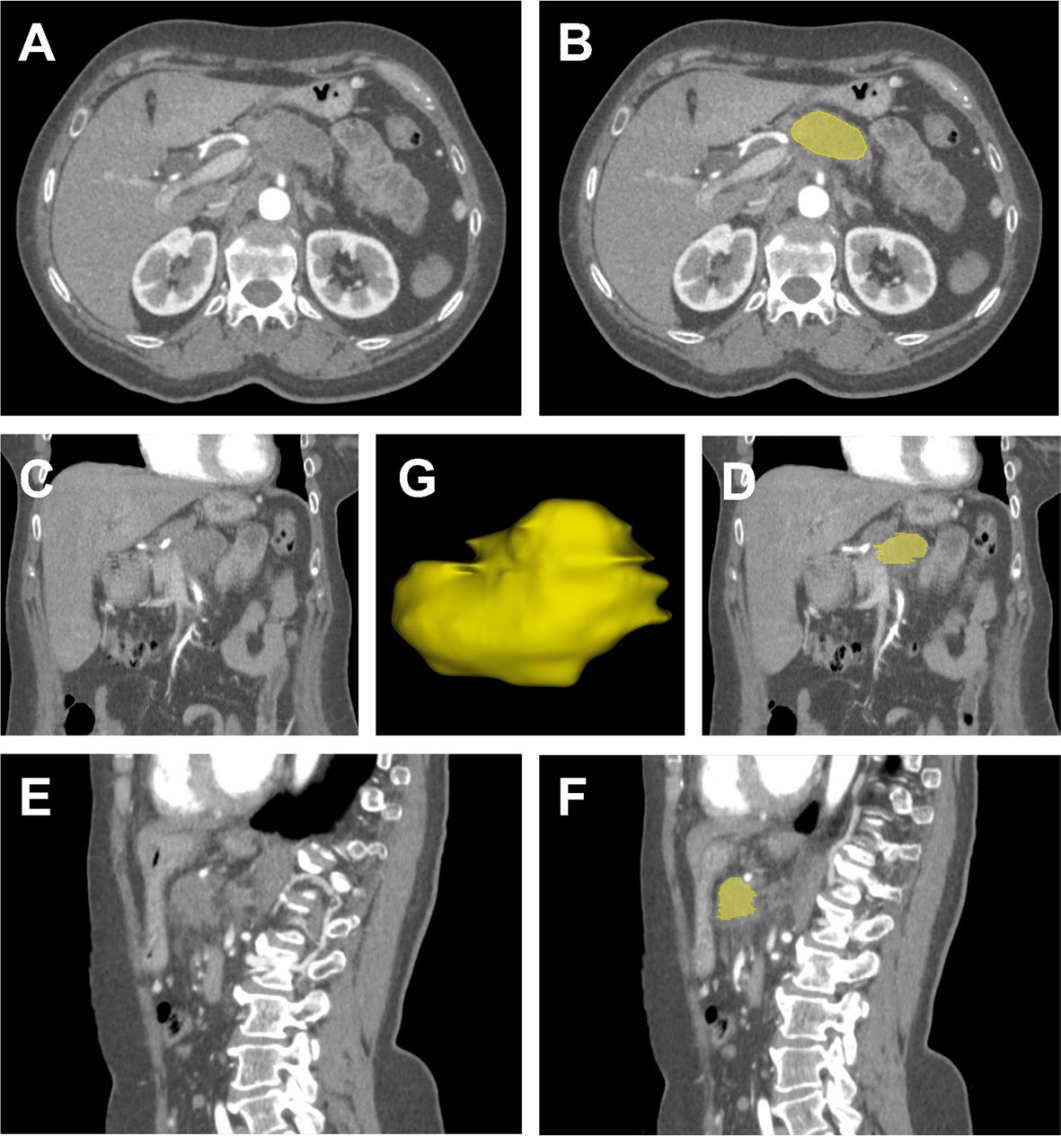
Case of a 50-year-old female who was admitted to the emergency department with severe abdominal pain radiating to the back and a weight loss of 12 kg. Diffuse pain over the abdominal area was registered during the physical examination. After the detection of a space-occupying pancreatic mass lesion by ultrasound, dual-energy computed tomography was performed. Transversal, coronal, and sagittal planes of contrast-enhanced scans (**A**, **C**, **E**) showed a mass of the pancreatic body which was highly suspicious of cancer. Histopathological examination revealed a pancreatic ductal adenocarcinoma. The right column shows the tumor outlined with a semi-automatic delineation method in yellow (**B**, **D**, **F**). A three-dimensional illustration of the tumor is displayed at the center of the image (**G**)

**Fig. 3:**
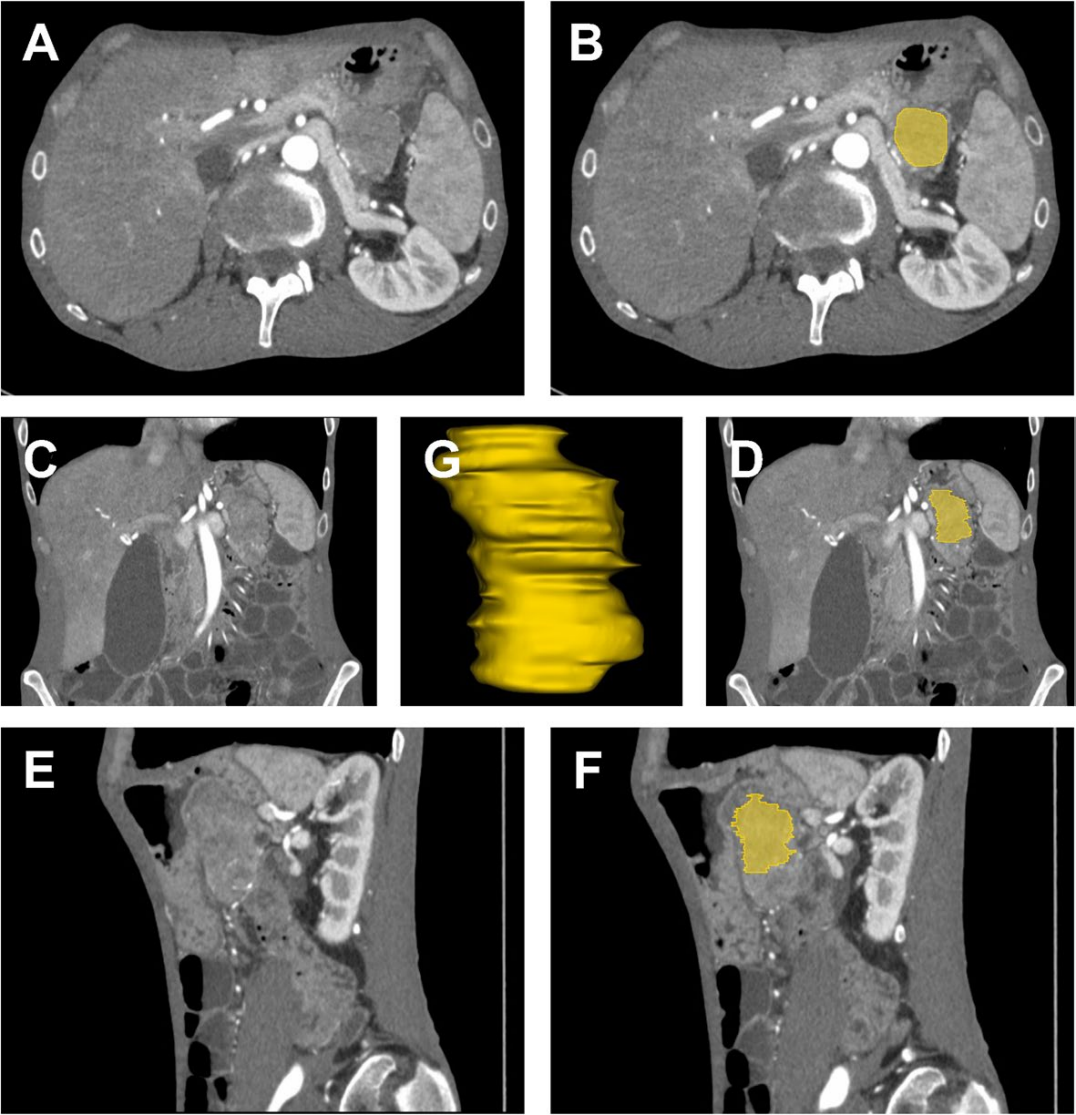
Case example of a 55-year-old male suffering from beltlike abdominal pain with metabolic acidosis (pH 7.3), hypokalemia (2.8 mmol/l), weight loss, and diarrhea for the past three months. After induced infusion therapy to compensate for the electrolyte imbalance, dual-energy computed tomography was performed, showing a hypodense mass of approximately 3.3 × 3.4 × 6.6 cm located in the pancreatic tail with occasional calcifications and finally proven ductal adenocarcinoma (**A**, **C**, **E**). Additionally to tumor segmentation in different planes (**B**, **D**, **F**), the pancreatic mass is also shown in 3D (**G**)

Two experienced radiologists (M.H.A. and V.K. with 6 and 4 years of radiomics experience, respectively) performed image segmentation and analysis. After the upload of CT datasets into 3D slicer software (3D slicer 4.6.2., Harvard University, Cambridge, USA), each segmentation was accomplished by a semi-automatic method for pancreatic structure assessment using the interactive segmentation algorithm GrowCut, as previously described [19–21]. After data upload, the first reliable 3D segmentation model of the pancreatic formation was obtained by adding a small subset of label points with the automated reconstruction of the remaining image. The algorithm iteratively labeled all points using a weighted similarity score comprising all pixels in the adjacent tissue. After successful segmentation and visual inspection, areas with artifacts or calcifications were manually removed. Case examples are illustrated in Figs. 2 and 3.

Each segmentation was reviewed by one experienced radiologist (S.S.M., 6 years of experience in radiomics analysis). If the reviewer disagreed with the final result, the segmentation process was repeated under corrected conditions. All radiologists were blinded to clinical, laboratory, and histological data.

PyRadiomics extension package for 3D slicer software (Version 5.1.0–2022-05–20) was used to extract radiomics features from final segmentations, resulting in a total of 107 features per segmentation [22]. Extracted features could be classified into seven categories as follows: Gray-Level Dependence Matrix (GLDM), Gray-Level Co-Occurrence Matrix (GLCM), Grey-Level Run Length Matrix (GLRLM), Gray-Level Size Zone Matrix (GLSZM), Neighboring Gray Tone Difference Matrix (NGTDM), Shape, and First Order.

For DECT-IC and fat fraction assessment, three circular region of interests (ROIs) of 1 cm^2^ per patient were placed manually within malignant, inflamed, or normal pancreatic tissue, strictly excluding adjacent vessels, calcifications, fat, or cystic formations. Fat quantification was performed using three-material decomposition with fat, pancreatic tissue, and iodine as the basis triplet. A commercially available material decomposition algorithm (Syngo vB15, Siemens Healthineers) available for research was used for the quantification of iodine uptake and fat.

The hardware platform for image processing was a standard computer (Apple MacBook Pro 16 “, M1 Pro CPU, 32 GB RAM, macOS Monterey 12.3.1., Apple Inc., Cupertino, USA).

### MRI scan protocol

All MR examinations were performed on a 3-Tesla MRI system (Magnetom Prisma, Siemens Healthineers) using a multichannel body surface coil. The protocol included a diffusion-weighted sequence with the following scan parameters: repetition time/echo time (msec), 6000/52; slice thickness, 4 mm; intersection gap, 4.8 mm; matrix, 280 × 248; flip angle, 90°. Apparent diffusion coefficients (ADC) were calculated with dedicated software (Syngo vB15, Siemens Healthineers) using *b*-values of 1000 s/mm^2^. Images were acquired in axial, coronal, and sagittal planes. For quantitative analysis of ADC maps, three circular ROIs of 1.5 cm^2^ were placed in suspected target lesions.

### Quantitative assessment of radiomics features

Initial analysis of datasets was performed by applying Euclidean distance matrices and low dimensional embedding with t-distributed stochastic neighbor embedding to investigate cluster distributions. All acquired features were randomly divided into training (60%) and test (40%) datasets, potentially resulting in significant variability between groups.

All analyses were performed using open-source packages in Python 3.9.13. and MedCalc (Version 20.123; Ostend, Belgium) [23]. The Quality Radiomics Score (1.0) yielded a value of 24 (https://radiomics.world/rqs) [24, 25].

### Statistical analysis

Analyses were performed with MedCalc (Version 20.123). The normality of datasets was evaluated using the Kolmogorov–Smirnov test. Normally distributed values were illustrated as mean ± standard deviation (SD), otherwise as median with interquartile range (IQR). A *P *value < 0.05 was considered statistically significant.

Comparisons between categorical and continuous variables were performed using chi-square statistic tests, one-way ANOVA, or two-tailed Student’s t-test, where appropriate. The Cox proportional hazards model was used to determine independent factors of CT texture analysis and DWI on overall survival. Overall survival was defined as the time from imaging until either death from any cause or the date that the patient was last known to be alive. All participants were followed up from the time of initial diagnosis. Multivariate Cox proportional hazards models were created by adjusting significant univariate prognostic parameters for clinically important confounders, such as sex, age, and tumor size. Findings from the Cox proportional hazards models were reported as hazard ratios with corresponding 95% confidence intervals (CIs). Receiver operating characteristic (ROC) curve analysis was performed to compare the accuracy of survival models. Areas under the ROC curves (AUCs) were measured for performance assessment of each model in the evaluation of tissue discrimination and survival. The reproducibility of measurements was evaluated by calculating intra-class correlation coefficients (ICC) for each radiomics feature. In this context, values below 0.5 indicate poor reliability, between 0.5 and 0.75 moderate reliability, between 0.75 and 0.9 good reliability, and any value above 0.9 excellent reliability [26].

## Results

**Fig. 4:**
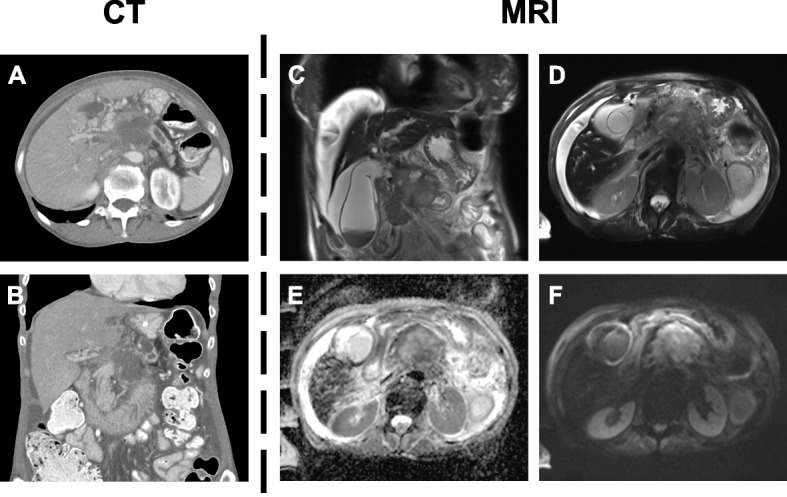
Illustration of a 74-year-old female who presented with typical symptoms of pancreatic cancer, having changes in her appetite, weight loss, and jaundice. Carbohydrate antigen 19–9 (CA 19–9) was considerably increased at approximately 11,000 U/mL, pointing towards advanced disease. After the initial diagnostic workup, the patient underwent dual-energy computed tomography (**A**, **B**) that identified a large pancreatic mass with consecutive ductal obstruction. One month later additional magnetic resonance imaging with T2-weighted sequences (**C**, **D**) confirmed the diagnosis and showed associated diffusion restriction in apparent diffusion coefficient maps (**E**) and diffusion-weighted imaging (**F**). Histopathology revealed the presence of pancreatic ductal adenocarcinoma

**Fig. 5:**
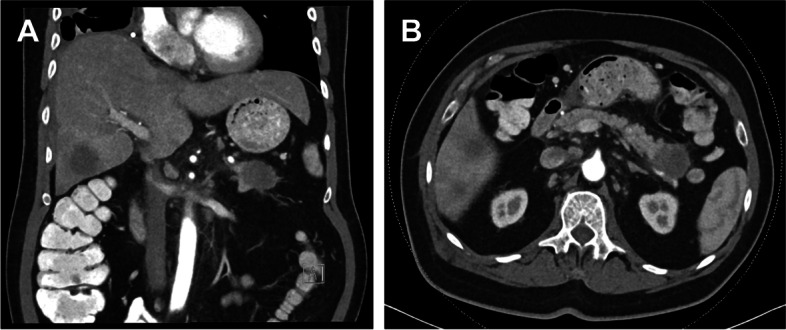
Images of a 61-year-old male who was referred to the gastrointestinal outpatient clinic with ten months' history of weight loss. More recently, he had developed epigastric pain, abdominal distension, and night sweats. His medication contained only pantoprazole and occasionally paracetamol. He drank half a liter wine every evening and smoked 20 cigarettes per day. A large mass in the tail of pancreas has been detected in the dual-energy computed tomography scan (**A**, **B**). The histopathological assessment revealed the presence of a pancreatic ductal adenocarcinoma

A total of 83 participants (65 ± 11 years; range, 34–87 years) with histologically proven pancreatic cancer were included, consisting of 52 men and 31 women (Table 1). Among these, 79 participants (95%) were diagnosed with pancreatic ductal adenocarcinoma, 3 with pancreatic neuroendocrine tumor (4%), and 1 with colloid carcinoma (1%). The most common localization was the pancreatic head, representing 65% of cases, followed by the pancreatic body (22%) and tail (13%). Two case examples of a large pancreatic mass lesion with ductal obstruction are presented in Figs. 4 and 5. The overall tumoral extension was 1.8 ± 2.9 cm^3^ (range, 1.1–8.1 cm^3^).

**Table 1:** Baseline characteristics of the study population (significant *P* values are written in bold)

***Variables*** ***- n (%) or mean (± SD)***	***Pancreatic cancer*** ***(n = 83)***	***Normal parenchyma*** ***(n = 40)***	***Pancreatitis*** ***(n = 20)***	***P value***
***Demographics***				
*Overall age (years)*	65 ± 11	64 ± 12	49 ± 16	** < 0.0001**
*Men (years)*	65 ± 12	63 ± 11	48 ± 14	** < 0.0001**
*Women (years)*	66 ± 10	64 ± 14	53 ± 20	** < 0.0001**
*Male sex (n)*	52 (63%)	29 (72%)	14 (70%)	
*Female sex (n)*	31 (37%)	11 (28%)	6 (30%)	
*BMI (kg/m*^*2*^*)*	26 ± 5	23 ± 4	24 ± 4	**0.0008**
***Vital signs***				
*Heart rate (bpm)*	79 ± 16	77 ± 17	81 ± 20	0.4748
*Systolic blood pressure (mmHg)*	136 ± 23	127 ± 18	122 ± 21	**0.0403**
*Diastolic blood pressure (mmHg)*	86 ± 13	80 ± 9	81 ± 6	**0.0086**
*Saturation of peripheral oxygen (%)*	98 ± 3	99 ± 2	98 ± 2	0.8040
*Temperature (°C)*	36.8 ± 0.6	36.7 ± 0.4	37.8 ± 0.6	0.6070
***T stage***				
*T1 (n)*	5 (6%)	-	-	
*T2 (n)*	26 (31%)	-	-	
*T3 (n)*	11 (13%)	-	-	
*T4 (n)*	41 (50%)	-	-	
***N stage***				
*N0 (n)*	13 (16%)	-	-	
*N1 (n)*	37 (45%)	-	-	
*N2 (n)*	33 (39%)	-	-	
***M stage***				
*M0 (n)*	38 (46%)	-	-	
*M1 (n)*	45 (54%)	-	-	
***Lymphovascular invasion (n)***	64 (77%)	-	-	
***Perineural invasion (n)***	27 (33%)	-	-	
***Risk factors***				
*Arterial hypertension (n)*	45 (54%)	8 (20%)	5 (25%)	
*Smoking (n)*	21 (25%)	10 (25%)	7 (35%)	
*Obesity (n)*	14 (17%)	4 (10%)	2 (10%)	
*Alcohol (n)*	12 (15%)	-	5 (25%)	
*Hypercholesterolemia (n)*	9 (11%)	5 (13%)	5 (25%)	
*Diabetes mellitus (n)*	7 (8%)	4 (10%)	3 (15%)	
*Chronic pancreatitis (n)*	4 (5%)	-	4 (20%)	
*Family history (n)*	3 (4%)	5 (13%)	-	

*Abbreviations: BMI* Body mass index, *SD* Standard deviation

According to the T stage classification, 5 participants (6%) had T1 stage, 26 (31%) T2, 11 (13%) T3, and 41 (50%) T4 stage. 13 participants (16%) showed no evidence of lymph node metastasis (N0), 37 participants (45%) had N1, and 33 (39%) N2 stage. A total of 45 participants (54%) had distant metastasis (M1).

Participants were followed up over 14 ± 12 months (range, 10–44 months).

### Comparative groups with normal or inflamed pancreatic parenchyma

A total of 40 participants (64 ± 12 years; range, 33–84 years) served as the comparative group without evidence of pancreatic pathologies. Another 20 participants (49 ± 16 years; range, 22–78 years) received acute biliary pancreatitis as their final diagnosis.

### Quantitative imaging characteristics

CT attenuation values of pancreatic tumor tissue (29.7 ± 15.3 HU) differed significantly from normal pancreatic parenchyma (37.4 ± 8.7 HU, *P* = 0.004) but not from inflamed pancreatic parenchyma (34.5 ± 11.2 HU, *P* = 0.19). HU values of neuroendocrine tumors (35.2 ± 6.3 HU) were not increased compared to pancreatic adenocarcinoma (29.5 ± 15.4 HU, *P* = 0.53). Fat fraction was significantly different between pancreatic cancer (16.5 ± 12.5%) and normal parenchyma (11.1 ± 4.0%, *P* = 0.008) without differences compared to inflamed parenchyma (13.0 ± 7.2%, *P* = 0.23). Iodine density of malignant tissue (0.8 ± 0.6 mg/mL) showed significant differences from both normal (2.6 ± 0.8 mg/mL, *P* < 0.001) and inflamed pancreatic tissue (1.8 ± 0.7 mg/mL, *P* < 0.001). Regarding DWI findings, ADC values from malignant pancreatic tissue (1.284 ± 0.245 mm^2^/s) were lower than those measured of normal parenchyma (1.676 ± 0.249 mm^2^/s, *P* < 0.001) and higher than those of inflamed tissue (1.134 ± 0.126 mm^2^/s, *P* = 0.01). Quantitative values of DECT variables and ADC mapping are presents in Table 2.

**Table 2:** Quantitative evaluation of CT iodine uptake and ADC mapping including patients with pancreatic cancer, normal pancreatic tissue, and pancreatitis (significant *P* values are written in bold)

***Variables*** ***- mean (± SD)***	***Pancreatic cancer*** ***(n = 83)***	***Normal parenchyma*** ***(n = 40)***	***Pancreatitis*** ***(n = 20)***	***P value***
***DECT***				
*Mean attenuation* *(HU)*	29.7 ± 15.3	37.4 ± 8.7	34.5 ± 11.2	**0.0100**
*Iodine uptake* *(mg/mL)*	0.8 ± 0.6	2.6 ± 0.8	1.8 ± 0.7	** < 0.0001**
*Fat fraction* *(%)*	16.5 ± 12.5	11.1 ± 4.0	13.0 ± 7.2	**0.0172**
***MRI ADC mapping***				
*ADC (10*^*−3*^* mm*^*2*^*/s)*	1.284 ± 0.245	1.676 ± 0.249	1.134 ± 0.126	** < 0.0001**

*Abbreviations: ADC* Apparent diffusion coefficient, *DECT* Dual-energy CT, *HU* Hounsfield Unit, *SD* Standard deviation

Values of malignant pancreatic tissue differed significantly from normal and inflamed tissue regarding first-order radiomics features (*P* < 0.001, respectively), GLCM (*P* < 0.001, respectively), GLDM (*P* < 0.001, respectively), GLRLM (*P* < 0.001, respectively), GLSZM (*P* < 0.001, respectively), NGTDM (*P* < 0.001, respectively), and shape (*P* < 0.001, respectively). ICC analysis was high to excellent for all feature classes, ranging from 0.84 (GLCM) to 0.98 (NGTDM). A list of extracted texture parameters is provided in Supplemental Tables 1 and 2.

The mean segmentation time using 3D slicer segmentation software was 8 min (range, 6–11 min).

### Diagnostic performance of CT-, MRI-, and radiomics features

Compared to non-cancer participants, the overall diagnostic performance of CT radiomics features, DECT-IC, and DWI to differentiate malignant parenchyma from normal pancreatic tissue was excellent (AUC 0.999, 95% CI, 0.962–1.0; *P* < 0.001). More specifically, the AUCs for radiomics features, DECT-IC, and DWI were 0.999 (95% CI, 0.969–1.0; *P* < 0.001), 0.974 (95% CI, 0.928–0.994; *P* < 0.001) and 0.862 (95% CI, 0.785–0.919; *P* < 0.001), respectively. Positive and negative predictive values are illustrated in Supplemental Table 1. If comparing cancer participants with the comparative normal group showing unspecific symptoms and normal pancreatic parenchyma, maximum PPV, as computed by using C-statistics, was 99% (95% CI, 92–100%) comprising all CT texture radiomics features, 96% (95% CI, 89–99%) for DECT-IC, 86% (95% CI, 79–91%) for DWI, and 99% (95% CI, 97–100%) for all three techniques together. Bivariate correlation analysis showed a significant correlation of radiomics features with DECT-IC (*r* = 0.74, 95% CI, 0.65–0.81; *P* < 0.001), fat fraction (*r* = 0.23, 95% CI, 0.05–0.39; *P* = 0.01), and mean attenuation (*r* = 0.24, 95% CI, 0.07–0.40; *P* = 0.007) when comprising all subjects with cancer. The diagnostic performance of all three techniques to discriminate between tumorous and normal pancreatic parenchyma was not affected after dichotomization of participants according to age above (≥ 50 years) and below 50 years (interaction *P* = 0.21).

**Fig. 6:**
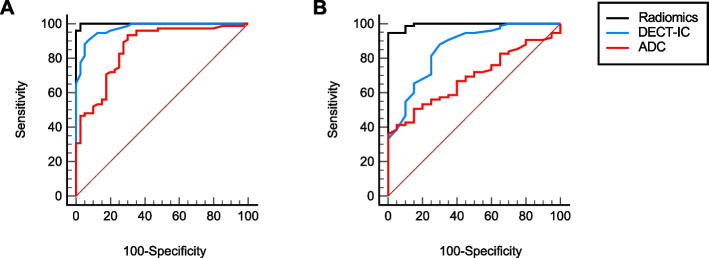
ROC analysis showing the diagnostic performance of radiomics (black line), DECT-IC (blue line), and MRI (red line) for detecting 1) malignant vs. normal pancreatic parenchyma (**A**), and 2) malignant vs. inflamed pancreatic parenchyma (**B**). ROC curves are depicted in black, blue, and red. *Abbreviations:DECT-IC*, Dual-energy CT-derived iodine concentration. *ROC*, Receiver operating characteristic curve. *ADC*, Apparent diffusion coefficient

The performance to distinguish between malignant and inflamed pancreatic tissue ranged between an AUC of 0.995 (95% CI, 0.955–1.0; *P* < 0.001) for radiomics features, 0.852 (95% CI, 0.767–0.914; *P* < 0.001) for DECT-IC, and 0.690 (95% CI, 0.587–0.780; *P* = 0.001) for DWI, respectively (Fig. 6). Overall AUC was 0.996 (95% CI, 0.894–1.0; *P* < 0.001) (Supplemental Table 2). The addition of CT texture features to DWI and iodine density measurements significantly increased the tumor detection rate to a diagnostic accuracy of 100%. The AUC improved by 0.307 (SE 0.056, *P* < 0.001) and by 0.216 (SE 0.061, *P* = 0.001), respectively, if adding radiomics features and iodine uptake to ADC.

The discriminative power of radiomics, DECT-IC, and DWI to differentiate malignant from normal pancreatic parenchyma was higher compared to the performance in malignant versus inflamed pancreatic parenchyma (*P* ≤ 0.02) (Supplemental Table 3).

### Prognostic value of imaging features in predicting all-cause mortality

During a follow-up of 14 ± 12 months (range, 10–44 months), a total of 51 deaths (61%) occurred among cancer patients. In Cox regression, radiomics features in their entirety were found to provide independent prognostic information for death (c-index = 0.709 [95% CI, 0.594–0.823], *P* = 0.02), even after adjustment for potential confounders that had been identified in the univariate analysis (Table 3). DECT-IC and DWI did not contribute to the prediction of outcomes (*P* = 0.59 and *P* = 0.16, respectively). Nevertheless, the combination of all three techniques showed a moderate overall prognostic power (c-index = 0.778 [95% CI, 0.697–0.864], *P* = 0.01).

**Table 3:** Performance of different cox-regression models to predict outcome combining radiomics features with clinical parameters (significant *P* values are written in bold)

	***Endpoint*** ***(all-cause mortality)***					
***Model***	***b***	***SE***	***Exp(b)***	***95% CI of Exp(b)***	***Chi-squared***	***P value***
*Unadjusted ****Model 1***	1.256	0.593	3.513	1.099 to 11.224	5.088	**0.0241**
*Adjusted ****Model 2***	1.434	0.615	4.196	1.258 to 13.995	11.207	**0.0037**
*Adjusted ****Model 3***	2.173	0.702	8.788	2.220 to 34.797	23.444	** < 0.0001**
*Adjusted ****Model 4***	2.415	0.739	11.192	2.630 to 47.627	29.014	** < 0.0001**
*Adjusted ****Model 5***	1.638	0.638	5.145	1.474 to 17.952	14.632	**0.0055**

Model 1: unadjusted radiomics model. Model 2: additionally adjusted by T stage. Model 3: additionally adjusted by alcohol abuse and smoking. Model 4: additionally adjusted by T stage, alcohol abuse, and smoking. Model 5: additionally adjusted by TNM stage

• Variables that did not reach univariate significance:

◦ Death: age (*P* = .75), sex (*P* = .29), arterial hypertension (*P* = .35), diabetes mellitus (*P* = .60), family history (*P* = .44), hypercholesterolemia (*P* = .75), chronic pancreatitis (*P* = .26), obesity (*P* = .21), C-reactive protein (*P* = .77), lipase (*P* = .44), lactate dehydrogenase (*P* = .47), creatinine (*P* = .49), glomerular filtration rate (*P* = .61), leucocytes (*P* = .99), CA 19–9 (*P* = .58), CEA (*P* = .76), N stage (*P* = .54), M stage (*P* = .53)

• Variables that reached univariate significance:

◦ Death: alcohol abuse (*P* = .04), smoking (*P* = .03), T stage (*P* = .03)

*Abbreviations: b* Regression coefficient, *SE* Standard error, *Exp(b)* Ratio of hazard rates, *CI* Confidence interval

Imaging features differed in the train/ test splits, with GLRLM (c-index = 0.719 [95% CI, 0.638–0.800], *P* < 0.001) and GLDM (c-index = 0.735 [95% CI, 0.644–0.827], *P* = 0.001) being the top-ranked features as part of the gray level matrix.

On multivariate analysis, alcohol abuse, smoking, and T stage were identified as independent prognostic parameters for poor outcome, showing a c-index ranging between 0.577 (95% CI, 0.510–0.644) and 0.608 (95% CI, 0.532–0.684) (*P* ≤ 0.04). Adding clinical features to quantitative radiomics biomarkers increased the Chi^2^ value significantly from 5.088 (c-index 0.709 [95% CI, 0.594–0.823], *P* = 0.02) to 29.014 (c-index 0.767 [95% CI, 0.678–0.856], *P* < 0.001).

## Discussion

Rapid and reliable detection of malignant lesions in patients with symptoms suggestive of pancreatic cancer remains often challenging due to small lesion size or insufficiently definable tumor margins in case of low image quality. This study demonstrated that a multiparametric approach involving DECT-IC, DWI, and radiomics can improve the discrimination of malignant lesions from inflamed or normal pancreatic parenchyma with higher diagnostic accuracy than every single modality (overall AUC ≥ 0.996, *P* < 0.001). Moreover, this approach allowed for the prediction of survival at moderate overall prognostic power.

Over the last decade, an increasing body of evidence suggests the incorporation of quantitative imaging biomarkers into established models of clinical decision-making to provide mineable tissue information by automated extraction of valuable imaging features [27]. As tumorous tissue typically shows heterogeneous features, which vary spatially and over time, characteristics of tumor heterogeneity might be of outstanding importance in the outcome prediction of cancer patients [28].

The present study has come to several important findings. First, we analyzed differences in texture parameters between tumorous and normal or inflamed pancreatic tissue using standard-of-care CT- and MRI examinations. Nearly all investigated features of malignant tissue showed a deviation from normal or inflamed pancreatic parenchyma. Most studies reporting on the texture analysis of tumorous tissue used GLCM to discriminate changes in microenvironment [29–31]. Higher tumor heterogeneity as indicated by dissimilarity and entropy is usually associated with greater variability of quantitative imaging parameters [14]. In line with previously published radiomics studies [14, 29, 32, 33], our results of high GLCM values reflected heterogeneous pancreatic tumor tissue associated with advanced TNM staging and a higher percentage of participants with distant metastases. We found that many parameters of textural analysis were stronger associated with overall survival than multiple classic parameters, such as tumor size. Using radiomics alone allowed us to correctly identify all 83 cases of malignant pancreatic mass lesions with high diagnostic accuracy.

Prediction of survival remains difficult and careful consideration of surgery is needed to avoid a highly morbid procedure for relatively little gain. With the advent of next-generation CT- and MR devices including various hard- and software improvements, imaging modalities increasingly attracted scientific attention in the evaluation of tumor characteristics and surgical resectability. Tumor heterogeneity can be assessed by either analyzing histological or imaging data. In this context, CT offers several advantages, such as non-invasiveness, general availability, and ease of use. As the routinely performed CT is usually part of a standard diagnostic procedure, its combination with MRI opens new perspectives in terms of a multiparametric approach for the assessment of pancreatic mass lesions.

All examined pancreatic lesions showed diffusion restriction, which was depicted as a low signal on ADC mapping and a high *b*-value on DWI. However, DWI- and ADC maps in our study could not reliably distinguish malignant pancreatic lesions from inflamed parenchyma (AUC = 0.690), whereas sufficient discriminative power was observed when comparing against normal parenchyma (AUC = 0.862). Possible explanations could be difficulties in exactly separating malignant from inflamed tissue when contouring pancreatic lesions or a small tumor size.

The addition of CT texture features to DWI and iodine density measurements significantly increased the tumor detection rate to a diagnostic accuracy of 100% and improved outcome prediction. A multiparametric combination of CT radiomics features, iodine density measurements, and DWI provided independent prognostic value even after adjustment for potential confounders. During a median follow-up of 14 ± 12 months (range, 10–44 months), this approach was found to provide significant independent prognostic information for death (*P* = 0.02).

The potential of radiomics to predict outcomes of patients with different types of cancer has been shown in different previous studies [14, 15, 29, 34, 35]. However, little is known about the value of our reported multiparametric approach in outcome prediction. The performance of our best working model that combines all three diagnostic approaches showed a moderate overall prognostic power. Previous studies in this field revealed performances in the same dimensions using radiomics alone or combined with clinical features [15, 34, 35]. We further benchmarked the combined model against clinical features and selected laboratory values. Most features in the low-ranked area did not reach relevance in different test splits. Gray level and first-order features yielded the highest importance in most of our constructed models, complemented by other radiomics features for even higher performances. For example, adding GLCM to shape features resulted in a net benefit of 0.045 (AUC 0.953 [95% CI, 0.900–0.983] vs. 0.998 [95% CI, 0.967–1.0], SE 0.017; *P* < 0.01).

Our findings underline the usefulness and potential of radiomics to aid in clinical decision-making rather than being merely complex and unmanageable datasets of texture spots. Considering the number of diverse radiomics features, Welch et al. formulated several safeguards to refine research about radiomics and ensure reliable model construction by identifying signature features as surrogates of future tumor behavior [36]. Identifying appropriate features that might be able to aid in risk stratification seems to be essential to avoid poor outcomes. Regarding time effectiveness, segmentation analysis with 3D slicer took 8 min on average, highlighting the good integrability of the segmentation process into daily clinical routine. However, novel fully automated algorithms as part of standard imaging analysis tools might save time.

DECT also facilitates the extraction of the fat content from iodine maps, which has emerged as an objective, image-based biomarker of disease (i.e., the calculation of fat concentration within a voxel of interest using algorithms for material decomposition) [37]. Methods for fat quantification are becoming increasingly popular in research and clinical practice, but these methods vary depending on the manufacturer, phase, and reconstruction software being used. Besides their role as energy storage depots for triglycerides, adipose cells inherit important endocrine, metabolic, hematological, immune, and structural functions [38]. Derangements in fat composition are not only seen in obesity, but also in patients with pancreatic cancer. In this context, pancreatic steatosis is regarded as an independent risk factor for pancreatic cancer [39, 40]. Moreover, increased pancreatic fat content progressively correlates with the risk for pancreatic cancer [41, 42]. In a study with 68 cases of histologically proven pancreatic ductal adenocarcinoma, risk of developing cancer significantly correlated with higher fatty infiltration, which has been assessed by calculating pancreatic attenuation in non-contrast CT scans [43]. In accordance, cancer patients in our study showed higher values of fatty pancreatic degeneration compared to normal parenchyma (*P* = 0.01). Future trials are needed to investigate clinical and imaging biomarkers, to validate available imaging modalities, and to establish diagnostic thresholds for the identification and graduation of pancreatic steatosis. Ameliorating the adverse effects of fatty infiltration will reduce the risk of developing pre-malignant lesions or even pancreatic cancer and further elucidate pathophysiological aspects.

Several study limitations have to be addressed. First, the assessment of iodine uptake and DWI was performed by placing ROIs in the center of the malignant lesions not covering the entire tumor area. Therefore, lesion characteristics could deviate from whole-tumor analyses that also included tumor periphery. Second, tumor heterogeneity was assessed regarding imaging biomarkers, not considering genetic alterations of pathological tumor tissue. Future studies should focus on linking texture features of tumor heterogeneity with genetic analysis to investigate subtypes of pancreatic cancer. Finally, the study was performed at a single center to avoid variabilities associated with scanners from different manufacturers or different scanner generations.

## Conclusions

In conclusion, this study showed that a multiparametric approach allows for accurate diagnosis of pancreatic mass lesions, as well as prediction of all-cause mortality. Therefore, merging radiomics with established imaging modalities may have the potential to identify cancer patients by computational allocation in specific survival models.

## Supplementary Information

**Additional file 1: Supplemental Table 1. **Discriminative power of dual-energy CT, MRI, and radiomics features to distinguish between malignant pancreatic tissue and normal parenchyma.

**Additional file 2: Supplemental Table 2. **Diagnostic performance of dual-energy CT, MRI, and radiomics features to discriminate between malignant and inflamed pancreatic tissue.

**Additional file 3:Supplemental Table 3. **Comparison of ROC curves malignant/normal vs. malignant/inflamed.

## Data Availability

All data generated or analyzed during this study are included in this published article.
