# Supplementary material for: Multiparametric detection and outcome prediction of pancreatic cancer involving dual-energy CT, diffusion-weighted MRI, and radiomics
Source: Cancer Imaging. 2023 Apr 18;23:38. doi: 10.1186/s40644-023-00549-8 (PMC10114410; doi:10.1186/s40644-023-00549-8)
Supplement: Supplementary file 1 — Additional file 1: Supplemental Table 1. Discriminative power of dual-energy CT, MRI, and radiomics features to distinguish between malignant pancreatic tissue and normal parenchyma. [file 40644_2023_549_MOESM1_ESM.doc]

**Supplemental Table 1.** Discriminative Power of Dual-Energy CT, MRI, and Radiomics Features to Distinguish Between Malignant Pancreatic Tissue and Normal Parenchyma.

| ***Parameter***  ***Malignant vs. Normal*** | ***Optimal***  ***threshold*** | *Sensitivity*  *(%)* | *Specificity (%)* | *AUC*  *overall* | *AUC overall*  *95% CI* | *PPV*  *(%)* | *NPV*  *(%)* | *P value*  *(AUC overall)* | *AUC*  *train* | *AUC train*  *95% CI* | *P value*  *(AUC train)* | *AUC*  *test* | *AUC test*  *95% CI* | *P value*  *(AUC test)* | *P value*  *(AUC overall vs. test)* |
| --- | --- | --- | --- | --- | --- | --- | --- | --- | --- | --- | --- | --- | --- | --- | --- |
|  |  |  |  |  |  |  |  |  |  |  |  |  |  |  |  |
| ***1) CT radiomics texture features*** |  |  |  |  |  |  |  |  |  |  |  |  |  |  |  |
|  |  |  |  |  |  |  |  |  |  |  |  |  |  |  |  |
| ***Radiomics Overall*** | 0.31 | 100 | 98 | 0.999 | 0.969-1.000 | 99 | 100 | <0.0001 | 0.998 | 0.964-1.000 | <0.0001 | 0.997 | 0.957-1.000 | <0.0001 | 0.9638 |
|  |  |  |  |  |  |  |  |  |  |  |  |  |  |  |  |
| ***First-order*** |  |  |  |  |  |  |  |  |  |  |  |  |  |  |  |
| *First-order Overall* | 0.54 | 96 | 100 | 0.996 | 0.963-1.000 | 100 | 91 | <0.0001 | 0.995 | 0.956-1.000 | <0.0001 | 0.996 | 0.952-1.000 | <0.0001 | 1.0000 |
| *10Percentile* | 24.00 | 46 | 78 | 0.584 | 0.491-0.672 | 81 | 41 | 0.1118 | 0.620 | 0.520-0.713 | 0.0297 | 0.638 | 0.530-0.737 | 0.0193 |  |
| *90Percentile* | 86.00 | 71 | 98 | 0.862 | 0.789-0.918 | 98 | 62 | <0.0001 | 0.882 | 0.804-0.936 | <0.0001 | 0.885 | 0.801-0.943 | <0.0001 |  |
| *Energy* | 7818644.00 | 57 | 93 | 0.694 | 0.605-0.774 | 94 | 51 | <0.0001 | 0.672 | 0.573-0.760 | 0.0011 | 0.669 | 0.562-0.765 | 0.0049 |  |
| *Entropy* | 2.02 | 83 | 98 | 0.945 | 0.889-0.978 | 99 | 74 | <0.0001 | 0.932 | 0.866-0.972 | <0.0001 | 0.930 | 0.856-0.973 | <0.0001 |  |
| *InterquartileRange* | 32.00 | 84 | 98 | 0.922 | 0.859-0.963 | 99 | 75 | <0.0001 | 0.908 | 0.836-0.956 | <0.0001 | 0.897 | 0.815-0.951 | <0.0001 |  |
| *Kurtosis* | 3.40 | 47 | 95 | 0.681 | 0.591-0.762 | 95 | 46 | 0.0001 | 0.657 | 0.558-0.747 | 0.0028 | 0.635 | 0.526-0.734 | 0.0232 |  |
| *Maximum* | 132.00 | 58 | 98 | 0.738 | 0.651-0.813 | 98 | 53 | <0.0001 | 0.762 | 0.669-0.839 | <0.0001 | 0.755 | 0.653-0.840 | <0.0001 |  |
| *Mean* | 53.50 | 60 | 93 | 0.761 | 0.676-0.833 | 94 | 53 | <0.0001 | 0.792 | 0.702-0.865 | <0.0001 | 0.805 | 0.708-0.881 | <0.0001 |  |
| *MeanAbsoluteDeviation* | 19.12 | 83 | 98 | 0.934 | 0.875-0.971 | 99 | 74 | <0.0001 | 0.920 | 0.851-0.964 | <0.0001 | 0.913 | 0.835-0.962 | <0.0001 |  |
| *Median* | 53.00 | 59 | 93 | 0.761 | 0.676-0.834 | 94 | 52 | <0.0001 | 0.792 | 0.702-0.865 | <0.0001 | 0.807 | 0.711-0.883 | <0.0001 |  |
| *Minimum* | -44.00 | 34 | 85 | 0.530 | 0.438-0.621 | 82 | 38 | 0.5765 | 0.553 | 0.453-0.650 | 0.3524 | 0.583 | 0.474-0.686 | 0.1725 |  |
| *Range* | 154.00 | 53 | 90 | 0.677 | 0.587-0.758 | 92 | 48 | 0.0002 | 0.669 | 0.571-0.758 | 0.0011 | 0.659 | 0.551-0.755 | 0.0064 |  |
| *RobustMeanAbsoluteDeviation* | 13.35 | 84 | 98 | 0.928 | 0.867-0.967 | 99 | 75 | <0.0001 | 0.913 | 0.842-0.959 | <0.0001 | 0.903 | 0.822-0.955 | <0.0001 |  |
| *RootMeanSquared* | 63.00 | 68 | 93 | 0.791 | 0.708-0.859 | 92 | 57 | <0.0001 | 0.820 | 0.733-0.888 | <0.0001 | 0.832 | 0.739-0.903 | <0.0001 |  |
| *Skewness* | -0.31 | 30 | 100 | 0.519 | 0.427-0.610 | 64 | 12 | 0.7137 | 0.501 | 0.402-0.600 | 0.9837 | 0.511 | 0.403-0.618 | 0.8604 |  |
| *TotalEnergy* | 11246540.49 | 64 | 100 | 0.821 | 0.742-0.884 | 100 | 57 | <0.0001 | 0.788 | 0.697-0.861 | <0.0001 | 0.756 | 0.654-0.840 | <0.0001 |  |
| *Uniformity* | 0.28 | 84 | 98 | 0.939 | 0.881-0.974 | 99 | 75 | <0.0001 | 0.925 | 0.856-0.967 | <0.0001 | 0.918 | 0.841-0.965 | <0.0001 |  |
| *Variance* | 574.97 | 83 | 98 | 0.937 | 0.879-0.973 | 99 | 74 | <0.0001 | 0.923 | 0.854-0.966 | <0.0001 | 0.916 | 0.839-0.964 | <0.0001 |  |
|  |  |  |  |  |  |  |  |  |  |  |  |  |  |  |  |
| ***GLCM*** |  |  |  |  |  |  |  |  |  |  |  |  |  |  |  |
| *GLCM Overall* | 0.33 | 100 | 98 | 0.998 | 0.967-1.000 | 99 | 100 | <0.0001 | 0.998 | 0.961-1.000 | <0.0001 | 0.997 | 0.954-1.000 | <0.0001 | 0.7902 |
| *Autocorrelation* | 21.06 | 69 | 75 | 0.708 | 0.619-0.786 | 85 | 54 | <0.0001 | 0.704 | 0.607-0.789 | 0.0001 | 0.694 | 0.587-0.786 | 0.0006 |  |
| *ClusterProminence* | 30.51 | 87 | 75 | 0.846 | 0.770-0.905 | 88 | 73 | <0.0001 | 0.825 | 0.739-0.892 | <0.0001 | 0.819 | 0.723-0.892 | <0.0001 |  |
| *ClusterShade* | -0.98 | 86 | 25 | 0.524 | 0.432-0.615 | 70 | 46 | 0.6713 | 0.539 | 0.439-0.637 | 0.502 | 0.563 | 0.454-0.667 | 0.3119 |  |
| *ClusterTendency* | 2.33 | 70 | 98 | 0.889 | 0.820-0.939 | 98 | 61 | <0.0001 | 0.871 | 0.792-0.929 | <0.0001 | 0.866 | 0.778-0.929 | <0.0001 |  |
| *Contrast* | 1.62 | 95 | 98 | 0.993 | 0.958-1.000 | 99 | 91 | <0.0001 | 0.991 | 0.949-1.000 | <0.0001 | 0.989 | 0.939-1.000 | <0.0001 |  |
| *Correlation* | 0.32 | 71 | 95 | 0.850 | 0.775-0.908 | 97 | 61 | <0.0001 | 0.827 | 0.740-0.893 | <0.0001 | 0.835 | 0.743-0.905 | <0.0001 |  |
| *DifferenceAverage* | 0.95 | 95 | 98 | 0.995 | 0.961-1.000 | 99 | 91 | <0.0001 | 0.993 | 0.953-1.000 | <0.0001 | 0.992 | 0.944-1.000 | <0.0001 |  |
| *DifferenceEntropy* | 1.70 | 95 | 98 | 0.992 | 0.956-1.000 | 98 | 91 | <0.0001 | 0.990 | 0.947-1.000 | <0.0001 | 0.987 | 0.936-0.999 | <0.0001 |  |
| *DifferenceVariance* | 0.70 | 95 | 98 | 0.991 | 0.954-1.000 | 99 | 91 | <0.0001 | 0.988 | 0.944-0.999 | <0.0001 | 0.985 | 0.932-0.999 | <0.0001 |  |
| *Id* | 0.61 | 99 | 95 | 0.996 | 0.962-1.000 | 98 | 97 | <0.0001 | 0.995 | 0.955-1.000 | <0.0001 | 0.993 | 0-947-1.000 | <0.0001 |  |
| *Idm* | 0.58 | 99 | 95 | 0.995 | 0.962-1.000 | 98 | 97 | <0.0001 | 0.994 | 0.955-1.000 | <0.0001 | 0.992 | 0.946-1.000 | <0.0001 |  |
| *Idmn* | 0.98 | 74 | 98 | 0.861 | 0.787-0.916 | 98 | 64 | <0.0001 | 0.859 | 0.777-0.919 | <0.0001 | 0.847 | 0,755-0.914 | <0.0001 |  |
| *Idn* | 0.90 | 90 | 93 | 0.937 | 0.878-0.973 | 96 | 82 | <0.0001 | 0.935 | 0.870-0.974 | <0.0001 | 0.936 | 0.863-0.976 | <0.0001 |  |
| *Imc1* | 0.07 | 76 | 98 | 0.921 | 0.858-0.962 | 98 | 66 | <0.0001 | 0.905 | 0.833-0.954 | <0.0001 | 0.908 | 0.828-0.958 | <0.0001 |  |
| *Imc2* | 0.41 | 74 | 73 | 0.746 | 0.659-0.820 | 85 | 57 | <0.0001 | 0.732 | 0.637-0.814 | <0.0001 | 0.742 | 0.638-0.828 | <0.0001 |  |
| *InverseVariance* | 0.47 | 83 | 98 | 0.886 | 0.816-0.936 | 99 | 74 | <0.0001 | 0.856 | 0.774-0.917 | <0.0001 | 0.821 | 0.725-0.893 | <0.0001 |  |
| *JointAverage* | 4.56 | 69 | 75 | 0.709 | 0.621-0.788 | 85 | 54 | <0.0001 | 0.706 | 0.609-0.791 | 0.0005 | 0.695 | 0.590-0.788 | 0.0005 |  |
| *JointEnergy* | 0.09 | 92 | 98 | 0.976 | 0.930-0.995 | 99 | 85 | <0.0001 | 0.969 | 0.916-0.993 | <0.0001 | 0.961 | 0.898-0.990 | <0.0001 |  |
| *JointEntropy* | 3.92 | 89 | 98 | 0.976 | 0.931-0.995 | 99 | 81 | <0.0001 | 0.970 | 0.917-0.993 | <0.0001 | 0.964 | 0.901-0.992 | <0.0001 |  |
| *MaximumProbability* | 0.18 | 88 | 98 | 0.968 | 0.920-0.992 | 99 | 80 | <0.0001 | 0.960 | 0.902-0.988 | <0.0001 | 0.948 | 0.880-0.984 | <0.0001 |  |
| *MCC* | 0.35 | 70 | 93 | 0.852 | 0.776-0.909 | 95 | 60 | <0.0001 | 0.833 | 0.747-0.898 | <0.0001 | 0.835 | 0.742-0.905 | <0.0001 |  |
| *SumAverage* | 9.12 | 69 | 75 | 0.709 | 0.621-0.788 | 85 | 54 | <0.0001 | 0.706 | 0.609-0.791 | 0.0005 | 0.695 | 0.590-0.788 | 0.0005 |  |
| *SumEntropy* | 2.62 | 72 | 98 | 0.904 | 0.837-0.949 | 98 | 63 | <0.0001 | 0.885 | 0.808-0.939 | <0.0001 | 0.881 | 0.796-0.940 | <0.0001 |  |
| *SumSquares* | 0.98 | 84 | 98 | 0.947 | 0.892-0.979 | 99 | 75 | <0.0001 | 0.935 | 0.869-0.974 | <0.0001 | 0.932 | 0.858-0.974 | <0.0001 |  |
|  |  |  |  |  |  |  |  |  |  |  |  |  |  |  |  |
| ***GLDM*** |  |  |  |  |  |  |  |  |  |  |  |  |  |  |  |
| *GLDM Overall* | 0.43 | 99 | 95 | 0.993 | 0.958-1.000 | 98 | 97 | <0.0001 | 0.991 | 0.949-1.000 | <0.0001 | 0.989 | 0.940-1.000 | <0.0001 | 0.6523 |
| *DependenceEntropy* | 5.86 | 45 | 98 | 0.661 | 0.570-0.744 | 97 | 46 | 0.0008 | 0.667 | 0.568-0.756 | 0.0016 | 0.683 | 0.576-0.777 | 0.0018 |  |
| *DependenceNonUniformity* | 124.06 | 59 | 95 | 0.781 | 0.698-0.851 | 96 | 53 | <0.0001 | 0.774 | 0.682-0.850 | <0.0001 | 0.768 | 0.667-0.851 | <0.0001 |  |
| *DependenceNonUniformityNormalized* | 0.08 | 93 | 98 | 0.984 | 0.942-0.998 | 99 | 87 | <0.0001 | 0.979 | 0.930-0.997 | <0.0001 | 0.982 | 0.929-0.998 | <0.0001 |  |
| *DependenceVariance* | 13.84 | 90 | 98 | 0.978 | 0.934-0.996 | 99 | 83 | <0.0001 | 0.972 | 0.920-0.994 | <0.0001 | 0.974 | 0.916-0.996 | <0.0001 |  |
| *GrayLevelNonUniformity* | 299.93 | 87 | 90 | 0.920 | 0.857-0.961 | 95 | 77 | <0.0001 | 0.914 | 0.843-0.960 | <0.0001 | 0.904 | 0.824-0,956 | <0.0001 |  |
| *GrayLevelVariance* | 1.01 | 83 | 98 | 0.941 | 0.884-0.975 | 99 | 74 | <0.0001 | 0.927 | 0.860-0.969 | <0.0001 | 0.924 | 0.848-0.969 | <0.0001 |  |
| *HighGrayLevelEmphasis* | 21.48 | 69 | 78 | 0.724 | 0.636-0.801 | 86 | 54 | <0.0001 | 0.719 | 0.623-0.803 | 0.0002 | 0.708 | 0.602-0.799 | 0.0002 |  |
| *LargeDependenceEmphasis* | 67.70 | 99 | 98 | 0.996 | 0.962-1.000 | 99 | 98 | <0.0001 | 0.995 | 0.955-1.000 | <0.0001 | 0.993 | 0.948-1.000 | <0.0001 |  |
| *LargeDependenceHighGrayLevelEmphasis* | 1815.90 | 66 | 95 | 0.798 | 0.716-0.865 | 97 | 58 | <0.0001 | 0.778 | 0.687-0.854 | <0.0001 | 0.785 | 0.685-0.864 | <0.0001 |  |
| *LargeDependenceLowGrayLevelEmphasis* | 3.93 | 94 | 88 | 0.951 | 0.896-0.982 | 94 | 88 | <0.0001 | 0.942 | 0.879-0.978 | <0.0001 | 0.931 | 0.858-0.974 | <0.0001 |  |
| *LowGrayLevelEmphasis* | 0.07 | 51 | 83 | 0.641 | 0.550-0.725 | 86 | 45 | 0.0046 | 0.638 | 0.539-0.730 | 0.0100 | 0.628 | 0.519-0.727 | 0.0317 |  |
| *SmallDependenceEmphasis* | 0.07 | 98 | 98 | 0.990 | 0.953-1.000 | 99 | 95 | <0.0001 | 0.987 | 0.943-0.999 | <0.0001 | 0.984 | 0.932-0.999 | <0.0001 |  |
| *SmallDependenceHighGrayLevelEmphasis* | 1.48 | 96 | 90 | 0.966 | 0.916-0.990 | 95 | 92 | <0.0001 | 0.958 | 0.900-0.987 | <0.0001 | 0.949 | 0.881-0.984 | <0.0001 |  |
| *SmallDependenceLowGrayLevelEmphasis* | 0.01 | 63 | 95 | 0.792 | 0.709-0.860 | 96 | 55 | <0.0001 | 0.778 | 0.686-0.853 | <0.0001 | 0.777 | 0.676-0.858 | <0.0001 |  |
|  |  |  |  |  |  |  |  |  |  |  |  |  |  |  |  |
| ***GLRLM*** |  |  |  |  |  |  |  |  |  |  |  |  |  |  |  |
| *GLRLM Overall* | 0.33 | 96 | 98 | 0.990 | 0.953-1.000 | 99 | 93 | <0.0001 | 0.988 | 0.943-0.999 | <0.0001 | 0.985 | 0.932-0.999 | <0.0001 | 0.6769 |
| *GrayLevelNonUniformity* | 197.45 | 86 | 88 | 0.899 | 0.832-0.946 | 93 | 75 | <0.0001 | 0.895 | 0.820-0.946 | <0.0001 | 0.885 | 0.801-0.943 | <0.0001 |  |
| *GrayLevelNonUniformityNormalized* | 0.26 | 82 | 98 | 0.938 | 0.880-0.974 | 97 | 72 | <0.0001 | 0.925 | 0.857-0.967 | <0.0001 | 0.920 | 0.843-0.967 | <0.0001 |  |
| *GrayLevelVariance* | 1.16 | 78 | 98 | 0.931 | 0.871-0.969 | 99 | 68 | <0.0001 | 0.916 | 0.846-0.961 | <0.0001 | 0.910 | 0.832-0.960 | <0.0001 |  |
| *HighGrayLevelRunEmphasis* | 21.71 | 69 | 78 | 0.725 | 0.637-0.801 | 86 | 54 | <0.0001 | 0.720 | 0.624-0.803 | 0.0002 | 0.707 | 0.602-0.798 | 0.0002 |  |
| *LongRunEmphasis* | 2.24 | 99 | 98 | 0.997 | 0.964-1.000 | 99 | 98 | <0.0001 | 0.996 | 0.957-1.000 | <0.0001 | 0.994 | 0.949-1.000 | <0.0001 |  |
| *LongRunHighGrayLevelEmphasis* | 63.36 | 60 | 90 | 0.722 | 0.634-0.799 | 93 | 52 | <0.0001 | 0.715 | 0.618-0.799 | <0.0001 | 0.727 | 0.623-0.816 | <0.0001 |  |
| *LongRunLowGrayLevelEmphasis* | 0.14 | 92 | 83 | 0.921 | 0.858-0.962 | 92 | 83 | <0.0001 | 0.908 | 0.836-0.956 | <0.0001 | 0.894 | 0.811-0.949 | <0.0001 |  |
| *LowGrayLevelRunEmphasis* | 0.07 | 51 | 83 | 0.643 | 0.551-0.727 | 83 | 44 | 0.0042 | 0.641 | 0.541-0.732 | 0.0089 | 0.628 | 0.519-0.727 | 0.0318 |  |
| *RunEntropy* | 3.34 | 54 | 93 | 0.656 | 0.565-0.739 | 94 | 49 | 0.0012 | 0.660 | 0.561-0.750 | 0.0026 | 0.679 | 0.572-0.774 | 0.0025 |  |
| *RunLengthNonUniformity* | 665.21 | 63 | 93 | 0.764 | 0.680-0.836 | 95 | 54 | <0.0001 | 0.758 | 0.665-0.837 | <0.0001 | 0.755 | 0.653-0.840 | <0.0001 |  |
| *RunLengthNonUniformityNormalized* | 0.62 | 99 | 95 | 0.993 | 0.958-1.000 | 98 | 97 | <0.0001 | 0.992 | 0.950-1.000 | <0.0001 | 0.990 | 0.942-1.000 | <0.0001 |  |
| *RunPercentage* | 0.75 | 99 | 98 | 0.995 | 0.960-1.000 | 99 | 98 | <0.0001 | 0.993 | 0.953-1.000 | <0.0001 | 0.992 | 0.944-1.000 | <0.0001 |  |
| *RunVariance* | 0.46 | 99 | 98 | 0.997 | 0.965-1.000 | 99 | 98 | <0.0001 | 0.996 | 0.958-1.000 | <0.0001 | 0.995 | 0.950-1.000 | <0.0001 |  |
| *ShortRunEmphasis* | 0.81 | 99 | 98 | 0.995 | 0.961-1.000 | 99 | 98 | <0.0001 | 0.994 | 0.954-1.000 | <0.0001 | 0.993 | 0.946-1.000 | <0.0001 |  |
| *ShortRunHighGrayLevelEmphasis* | 18.29 | 83 | 78 | 0.827 | 0.749-0.889 | 89 | 69 | <0.0001 | 0.820 | 0.733-0.888 | <0.0001 | 0.809 | 0.713-0.884 | <0.0001 |  |
| *ShortRunLowGrayLevelEmphasis* | 0.07 | 39 | 85 | 0.540 | 0.448-0.630 | 84 | 40 | 0.4387 | 0.539 | 0.439-0.637 | 0.4882 | 0.524 | 0.416-0.630 | 0.7039 |  |
|  |  |  |  |  |  |  |  |  |  |  |  |  |  |  |  |
| ***GLSZM*** |  |  |  |  |  |  |  |  |  |  |  |  |  |  |  |
| *GLSZM Overall* | 0.49 | 98 | 100 | 0.995 | 0.960-1.000 | 100 | 95 | <0.0001 | 0.993 | 0.953-1.000 | <0.0001 | 0.991 | 0.943-1.000 | <0.0001 | 0.6931 |
| *GrayLevelNonUniformity* | 15.42 | 65 | 90 | 0.777 | 0.693-0.847 | 93 | 55 | <0.0001 | 0.771 | 0.679-0.847 | <0.0001 | 0.770 | 0.669-0.852 | <0.0001 |  |
| *GrayLevelNonUniformityNormalized* | 0.22 | 57 | 95 | 0.821 | 0.741-0.884 | 96 | 51 | <0.0001 | 0.810 | 0.722-0.880 | <0.0001 | 0.811 | 0.715-0.886 | <0.0001 |  |
| *GrayLevelVariance* | 3.48 | 78 | 98 | 0.899 | 0.831-0.946 | 99 | 68 | <0.0001 | 0.877 | 0.799-0.933 | <0.0001 | 0.856 | 0.766-0.921 | <0.0001 |  |
| *HighGrayLevelZoneEmphasis* | 21.42 | 61 | 85 | 0.746 | 0.659-0.820 | 90 | 52 | <0.0001 | 0.735 | 0.640-0.817 | <0.0001 | 0.738 | 0.635-0.825 | <0.0001 |  |
| *LargeAreaEmphasis* | 5561.08 | 84 | 100 | 0.948 | 0.892-0.980 | 100 | 76 | <0.0001 | 0.939 | 0.875-0.977 | <0.0001 | 0.936 | 0.863-0.976 | <0.0001 |  |
| *LargeAreaHighGrayLevelEmphasis* | 103187.82 | 82 | 93 | 0.888 | 0.819-0.938 | 96 | 71 | <0.0001 | 0.877 | 0.799-0.933 | <0.0001 | 0.863 | 0.774-0.926 | <0.0001 |  |
| *LargeAreaLowGrayLevelEmphasis* | 284.82 | 90 | 98 | 0.982 | 0.940-0.998 | 99 | 83 | <0.0001 | 0.977 | 0.927-0.996 | <0.0001 | 0.974 | 0.916-0.996 | <0.0001 |  |
| *LowGrayLevelZoneEmphasis* | 0.14 | 46 | 83 | 0.587 | 0.494-0.675 | 84 | 42 | 0.0944 | 0.584 | 0.484-0.680 | 0.1308 | 0.572 | 0.463-0.676 | 0.2383 |  |
| *SizeZoneNonUniformity* | 22.88 | 51 | 78 | 0.562 | 0.470-0.652 | 82 | 43 | 0.2217 | 0.559 | 0.459-0.656 | 0.2952 | 0.591 | 0.483-0.694 | 0.1389 |  |
| *SizeZoneNonUniformityNormalized* | 0.27 | 77 | 75 | 0.806 | 0.725-0.872 | 87 | 61 | <0.0001 | 0.820 | 0.733-0.888 | <0.0001 | 0.788 | 0.689-0.867 | <0.0001 |  |
| *SmallAreaEmphasis* | 0.53 | 78 | 68 | 0.801 | 0.720-0.868 | 83 | 61 | <0.0001 | 0.818 | 0.731-0.887 | <0.0001 | 0.785 | 0.685-0.864 | <0.0001 |  |
| *SmallAreaHighGrayLevelEmphasis* | 11.73 | 71 | 83 | 0.816 | 0.736-0.880 | 89 | 58 | <0.0001 | 0.817 | 0.730-0.886 | <0.0001 | 0.803 | 0.705-0.879 | <0.0001 |  |
| *SmallAreaLowGrayLevelEmphasis* | 0.05 | 36 | 85 | 0.532 | 0.440-0.622 | 83 | 39 | 0.5415 | 0.539 | 0.439-0.637 | 0.6382 | 0.529 | 0.421-0.635 | 0.6382 |  |
| *ZoneEntropy* | 4.62 | 51 | 85 | 0.608 | 0.516-0.695 | 88 | 45 | 0.0305 | 0.625 | 0.525-0.717 | 0.0219 | 0.623 | 0.515-0.723 | 0.0457 |  |
| *ZonePercentage* | 0.06 | 94 | 98 | 0.986 | 0.945-0.999 | 98 | 91 | <0.0001 | 0.982 | 0.934-0.998 | <0.0001 | 0.977 | 0.920-0.997 | <0.0001 |  |
| *ZoneVariance* | 5380.13 | 84 | 100 | 0.940 | 0.883-0.975 | 100 | 76 | <0.0001 | 0.934 | 0.868-0.973 | <0.0001 | 0.930 | 0.856-0.973 | <0.0001 |  |
|  |  |  |  |  |  |  |  |  |  |  |  |  |  |  |  |
| ***NGTDM*** |  |  |  |  |  |  |  |  |  |  |  |  |  |  |  |
| *NGDTM Overall* | 0.51 | 93 | 95 | 0.979 | 0.935-0.996 | 98 | 86 | <0.0001 | 0.976 | 0.925-0.996 | <0.0001 | 0.968 | 0.908-0.994 | <0.0001 | 0.5490 |
| *Busyness* | 4.20 | 89 | 90 | 0.964 | 0.914-0.989 | 95 | 80 | <0.0001 | 0.957 | 0.898-0.987 | <0.0001 | 0.952 | 0.885-0.986 | <0.0001 |  |
| *Coarseness* | 0.01 | 77 | 88 | 0.835 | 0.757-0.896 | 93 | 65 | <0.0001 | 0.825 | 0.739-0.893 | <0.0001 | 0.815 | 0.719-0.889 | <0.0001 |  |
| *Complexity* | 18.12 | 82 | 83 | 0.840 | 0.763-0.900 | 91 | 69 | <0.0001 | 0.845 | 0.762-0.909 | <0.0001 | 0.824 | 0.729-0,896 | <0.0001 |  |
| *Contrast* | 0.03 | 86 | 98 | 0.939 | 0.881-0.974 | 96 | 77 | <0.0001 | 0.941 | 0.878-0.978 | <0.0001 | 0.933 | 0.860-0.975 | <0.0001 |  |
| *Strength* | 0.12 | 81 | 95 | 0.938 | 0.880-0.973 | 96 | 70 | <0.0001 | 0.922 | 0.854-0.966 | <0.0001 | 0.914 | 0.836-0.963 | <0.0001 |  |
|  |  |  |  |  |  |  |  |  |  |  |  |  |  |  |  |
| ***Shape*** |  |  |  |  |  |  |  |  |  |  |  |  |  |  |  |
| *Shape Overall* | 0.47 | 93 | 90 | 0.953 | 0.900-0.983 | 95 | 86 | <0.0001 | 0.953 | 0.893-0.985 | <0.0001 | 0.941 | 0.871-0.980 | <0.0001 | 0.6896 |
| *Elongation* | 0.72 | 43 | 68 | 0.515 | 0.423-0.606 | 74 | 37 | 0.7839 | 0.503 | 0.404-0.603 | 0.9519 | 0.505 | 0.397-0.612 | 0.9418 |  |
| *Flatness* | 0.57 | 60 | 63 | 0.578 | 0.486-0.666 | 76 | 43 | 0.1607 | 0.572 | 0.472-0.668 | 0.2179 | 0.552 | 0.443-0.657 | 0.4033 |  |
| *LeastAxisLength* | 9.51 | 80 | 98 | 0.884 | 0.814-0.935 | 99 | 70 | <0.0001 | 0.867 | 0.787-0.925 | <0.0001 | 0.850 | 0.759-0.916 | <0.0001 |  |
| *MajorAxisLength* | 16.51 | 86 | 95 | 0.942 | 0.884-0.976 | 97 | 76 | <0.0001 | 0.934 | 0.868-0.973 | <0.0001 | 0.925 | 0.850-0.970 | <0.0001 |  |
| *Maximum2DDiameterColumn* | 17.72 | 84 | 90 | 0.910 | 0.845-0.954 | 95 | 74 | <0.0001 | 0.899 | 0.825-0.949 | <0.0001 | 0.894 | 0.811-0.949 | <0.0001 |  |
| *Maximum2DDiameterRow* | 14.57 | 93 | 90 | 0.964 | 0.914-0.989 | 95 | 86 | <0.0001 | 0.957 | 0.899-0.987 | <0.0001 | 0.956 | 0.890-0.988 | <0.0001 |  |
| *Maximum2DDiameterSlice* | 19.14 | 83 | 95 | 0.927 | 0.866-0.966 | 97 | 73 | <0.0001 | 0.926 | 0.858-0.968 | <0.0001 | 0.913 | 0.834-0.962 | <0.0001 |  |
| *Maximum3DDiameter* | 22.67 | 82 | 98 | 0.938 | 0.880-0.973 | 99 | 72 | <0.0001 | 0.933 | 0.867 -0.972 | <0.0001 | 0.926 | 0.851-0.971 | <0.0001 |  |
| *MeshVolume* | 1481.13 | 83 | 98 | 0.936 | 0.878-0.972 | 99 | 74 | <0.0001 | 0.930 | 0.864-0.971 | <0.0001 | 0.919 | 0.843-0.966 | <0.0001 |  |
| *MinorAxisLength* | 11.87 | 84 | 95 | 0.936 | 0.877-0.972 | 97 | 75 | <0.0001 | 0.934 | 0.869-0.973 | <0.0001 | 0.921 | 0.844-0.967 | <0.0001 |  |
| *Sphericity* | 0.71 | 65 | 83 | 0.759 | 0.674-0.832 | 87 | 53 | <0.0001 | 0.746 | 0.652-0.826 | <0.0001 | 0.740 | 0.637-0.827 | <0.0001 |  |
| *SurfaceArea* | 590.54 | 92 | 90 | 0.936 | 0.877-0.972 | 95 | 84 | <0.0001 | 0.931 | 0.865-0.971 | <0.0001 | 0.922 | 0.846-0.968 | <0.0001 |  |
| *SurfaceVolumeRatio* | 0.60 | 78 | 100 | 0.903 | 0.836-0.949 | 100 | 69 | <0.0001 | 0.890 | 0.814-0.943 | <0.0001 | 0.885 | 0.800-0.942 | <0.0001 |  |
| *VoxelVolume* | 1501.49 | 84 | 98 | 0.936 | 0.878-0.972 | 99 | 75 | <0.0001 | 0.930 | 0.864-0.971 | <0.0001 | 0.919 | 0.843-0.966 | <0.0001 |  |
|  |  |  |  |  |  |  |  |  |  |  |  |  |  |  |  |
|  |  |  |  |  |  |  |  |  |  |  |  |  |  |  |  |
| ***2) CT iodine uptake*** |  |  |  |  |  |  |  |  |  |  |  |  |  |  |  |
| *Mean attenuation (HU)* | 29.80 | 48 | 90 | 0.684 | 0.594-0.765 | 91 | 46 | 0.0002 | 0.736 | 0.640-0.817 | <0.0001 | 0.741 | 0.637-0.828 | <0.0001 | 0.4261 |
| *Iodine uptake (mg/mL)* | 1.50 | 92 | 93 | 0.974 | 0.928-0.994 | 96 | 84 | <0.0001 | 0.972 | 0.920-0.994 | <0.0001 | 0.975 | 0.917-0.996 | <0.0001 | 0.9564 |
| *Fat fraction (%)* | 16.70 | 57 | 95 | 0.698 | 0.609-0.778 | 96 | 52 | <0.0001 | 0.750 | 0.655-0.829 | <0.0001 | 0.769 | 0.667-0.851 | <0.0001 | 0.3092 |
|  |  |  |  |  |  |  |  |  |  |  |  |  |  |  |  |
| ***3) MRI ADC Mapping*** |  |  |  |  |  |  |  |  |  |  |  |  |  |  |  |
| *ADC value (mm^2^/s)* | 1.616 | 94 | 70 | 0.862 | 0.785-0.919 | 86 | 85 | <0.0001 | 0.848 | 0.762-0.913 | <0.0001 | 0.834 | 0.736-0.906 | <0.0001 | 0.6323 |
|  |  |  |  |  |  |  |  |  |  |  |  |  |  |  |  |
| ***4) Overall radiomics, CT iodine uptake, and MRI ADC Mapping*** | 0.02 | 99 | 100 | 0.999 | 0.962-1.000 | 99 | 100 | <0.0001 | 0.999 | 0.963-1.000 | <0.0001 | 0.998 | 0.942-1.000 | <0.0001 | 0.9426 |
|  |  |  |  |  |  |  |  |  |  |  |  |  |  |  |  |
|  |  |  |  |  |  |  |  |  |  |  |  |  |  |  |  |

*Abbreviations: AUC, area under the curve. PPV, positive predictive value. NPV, negative predictive value. GLCM, Gray-Level Co-Occurrence Matrix. GLDM, Gray-Level Dependence Matrix. GLRLM, Grey-Level Run Length Matrix. GLSZM, Gray-Level Size Zone Matrix. NGTDM, Neighboring Gray Tone Difference Matrix. HU, Hounsfield unit. RU, relative unit. ADC, apparent diffusion coefficient.*
