# Supplementary material for: Multiparametric detection and outcome prediction of pancreatic cancer involving dual-energy CT, diffusion-weighted MRI, and radiomics
Source: Cancer Imaging. 2023 Apr 18;23:38. doi: 10.1186/s40644-023-00549-8 (PMC10114410; doi:10.1186/s40644-023-00549-8)
Supplement: Supplementary file 2 — Additional file 2: Supplemental Table 2. Diagnostic performance of dual-energy CT, MRI, and radiomics features to discriminate between malignant and inflamed pancreatic tissue. [file 40644_2023_549_MOESM2_ESM.doc]

**Supplemental Table 2.** Diagnostic Performance of Dual-Energy CT, MRI, and Radiomics Features to Discriminate Between Malignant and Inflamed Pancreatic Tissue.

| ***Parameter***  ***Malignant vs. Inflamed*** | ***Optimal threshold*** | *Sensitivity*  *(%)* | *Specificity (%)* | *AUC*  *overall* | *AUC overall*  *95% CI* | *PPV*  *(%)* | *NPV*  *(%)* | *P value*  *(AUC overall)* | *AUC*  *train* | *AUC train*  *95% CI* | *P value*  *(AUC train)* | *AUC*  *test* | *AUC test*  *95% CI* | *P value*  *(AUC test)* | *P value*  *(AUC overall vs. test)* |
| --- | --- | --- | --- | --- | --- | --- | --- | --- | --- | --- | --- | --- | --- | --- | --- |
|  |  |  |  |  |  |  |  |  |  |  |  |  |  |  |  |
| ***1) CT radiomics texture features*** |  |  |  |  |  |  |  |  |  |  |  |  |  |  |  |
|  |  |  |  |  |  |  |  |  |  |  |  |  |  |  |  |
| ***Radiomics Overall*** | 0.48 | 95 | 100 | 0.995 | 0.955-1.000 | 100 | 83 | <0.0001 | 0.994 | 0.948-1.000 | <0.0001 | 0.991 | 0.932-1.000 | <0.0001 | 0.6293 |
|  |  |  |  |  |  |  |  |  |  |  |  |  |  |  |  |
| ***First-order*** |  |  |  |  |  |  |  |  |  |  |  |  |  |  |  |
| *First-order Overall* | 0.62 | 88 | 95 | 0.960 | 0.901-0.989 | 99 | 66 | <0.0001 | 0.956 | 0.888-0.987 | <0.0001 | 0.954 | 0.875-0.990 | <0.0001 | 0.8635 |
| *10Percentile* | 37.00 | 35 | 90 | 0.632 | 0.531-0.725 | 94 | 25 | 0.0432 | 0.617 | 0.509-0.718 | 0.0820 | 0.577 | 0.453-0.694 | 0.2945 |  |
| *90Percentile* | 74.00 | 54 | 90 | 0.686 | 0.587-0.774 | 96 | 32 | 0.0024 | 0.690 | 0.584-0.784 | 0.0026 | 0.720 | 0.600-0.821 | 0.0010 |  |
| *Energy* | 20225819.00 | 35 | 95 | 0.570 | 0.469-0.668 | 97 | 26 | 0.2324 | 0.545 | 0.437-0.650 | 0.4720 | 0.554 | 0.430-0.673 | 0.4266 |  |
| *Entropy* | 2.08 | 86 | 95 | 0.935 | 0.869-0.974 | 99 | 61 | <0.0001 | 0.924 | 0.848-0.969 | <0.0001 | 0.917 | 0.826-0.970 | <0.0001 |  |
| *InterquartileRange* | 32.00 | 84 | 95 | 0.906 | 0.833-0.955 | 99 | 59 | <0.0001 | 0.889 | 0.805-0.945 | <0.0001 | 0.876 | 0.776-0.943 | <0.0001 |  |
| *Kurtosis* | 3.14 | 65 | 85 | 0.747 | 0.652-0.828 | 95 | 37 | <0.0001 | 0.721 | 0.617-0.811 | <0.0001 | 0.702 | 0.581-0.805 | 0.0009 |  |
| *Maximum* | 133.00 | 60 | 75 | 0.655 | 0.554-0.745 | 91 | 31 | 0.0048 | 0.666 | 0.559-0.762 | 0.0040 | 0.664 | 0.541-0.773 | 0.0114 |  |
| *Mean* | 50.50 | 53 | 70 | 0.540 | 0.439-0.638 | 88 | 26 | 0.5590 | 0.543 | 0.434-0.648 | 0.5383 | 0.579 | 0.455-0.696 | 0.2905 |  |
| *MeanAbsoluteDeviation* | 19.55 | 86 | 95 | 0.921 | 0.851-0.965 | 99 | 61 | <0.0001 | 0.906 | 0.827-0.958 | <0.0001 | 0.892 | 0.795-0.954 | <0.0001 |  |
| *Median* | 49.00 | 52 | 70 | 0.541 | 0.440-0.640 | 88 | 26 | 0.5460 | 0.546 | 0.438-0.653 | 0.5040 | 0.586 | 0.462-0.703 | 0.2485 |  |
| *Minimum* | 20.00 | 49 | 85 | 0.636 | 0.535-0.728 | 93 | 29 | 0.0254 | 0.617 | 0.509-0.718 | 0.1725 | 0.578 | 0.454-0.696 | 0.2637 |  |
| *Range* | 168.00 | 61 | 90 | 0.702 | 0.604-0.788 | 96 | 36 | 0.0001 | 0.694 | 0.588-0.787 | 0.0003 | 0.700 | 0.578-0.803 | 0.0012 |  |
| *RobustMeanAbsoluteDeviation* | 12.71 | 82 | 100 | 0.911 | 0.839-0.958 | 100 | 57 | <0.0001 | 0.895 | 0.813-0.950 | <0.0001 | 0.881 | 0.781-0,946 | <0.0001 |  |
| *RootMeanSquared* | 57.64 | 60 | 70 | 0.593 | 0.492-0.689 | 89 | 30 | 0.1519 | 0.598 | 0.489-0.700 | 0.1442 | 0.637 | 0.513-0.749 | 0,0548 |  |
| *Skewness* | 0.11 | 64 | 0 | 0.537 | 0.436-0.636 | 77 | 12 | 0.4988 | 0.522 | 0.414-0.629 | 0.7053 | 0.516 | 0.393-0.637 | 0.8138 |  |
| *TotalEnergy* | 11562161.14 | 64 | 90 | 0.743 | 0.648-0.824 | 96 | 38 | <0.0001 | 0.714 | 0.609-0.805 | <0.0001 | 0.687 | 0.565-0.793 | 0.0024 |  |
| *Uniformity* | 0.27 | 87 | 95 | 0.927 | 0.859-0.969 | 99 | 63 | <0.0001 | 0.914 | 0.835-0.962 | <0.0001 | 0.901 | 0.806-0.959 | <0.0001 |  |
| *Variance* | 574.97 | 83 | 95 | 0.923 | 0.854-0.967 | 99 | 58 | <0.0001 | 0.910 | 0.831-0.960 | <0.0001 | 0.899 | 0.804-0.958 | <0.0001 |  |
|  |  |  |  |  |  |  |  |  |  |  |  |  |  |  |  |
| ***GLCM*** |  |  |  |  |  |  |  |  |  |  |  |  |  |  |  |
| *GLCM Overall* | 0.57 | 94 | 100 | 0.992 | 0.950-1.000 | 100 | 80 | <0.0001 | 0.991 | 0.943-1.000 | <0.0001 | 0.987 | 0.925-1.000 | <0.0001 | 0.6440 |
| *Autocorrelation* | 19.42 | 60 | 85 | 0.672 | 0.573-0.762 | 94 | 34 | 0.0023 | 0.663 | 0.556-0.759 | 0.0060 | 0.664 | 0.541-0.773 | 0.0144 |  |
| *ClusterProminence* | 21.07 | 71 | 90 | 0.823 | 0.735-0.891 | 97 | 43 | <0.0001 | 0.806 | 0.709-0.882 | <0.0001 | 0.792 | 0.678-0.880 | <0.0001 |  |
| *ClusterShade* | -0.34 | 65 | 20 | 0.501 | 0.401-0.601 | 77 | 12 | 0.9860 | 0.516 | 0.408-0.622 | 0.8206 | 0.544 | 0.421-0.664 | 0.5521 |  |
| *ClusterTendency* | 2.66 | 78 | 90 | 0.873 | 0.794-0.931 | 97 | 50 | <0.0001 | 0.854 | 0.763-0.919 | <0.0001 | 0.842 | 0.735-0.918 | <0.0001 |  |
| *Contrast* | 1.23 | 92 | 100 | 0.987 | 0.941-0.999 | 100 | 74 | <0.0001 | 0.984 | 0.932-0.999 | <0.0001 | 0.978 | 0.911.0.998 | <0.0001 |  |
| *Correlation* | 0.30 | 74 | 90 | 0.805 | 0.716-0.877 | 95 | 44 | <0.0001 | 0.791 | 0.692-0.869 | <0.0001 | 0.789 | 0.675-0.877 | <0.0001 |  |
| *DifferenceAverage* | 0.81 | 92 | 100 | 0.989 | 0.944-1.000 | 100 | 76 | <0.0001 | 0.986 | 0.936-0.999 | <0.0001 | 0.981 | 0.915-0.999 | <0.0001 |  |
| *DifferenceEntropy* | 1.55 | 92 | 100 | 0.988 | 0.943-0.999 | 100 | 74 | <0.0001 | 0.986 | 0.934-0.999 | <0.0001 | 0.980 | 0.914-0.999 | <0.0001 |  |
| *DifferenceVariance* | 0.56 | 90 | 100 | 0.983 | 0.935-0.998 | 100 | 71 | <0.0001 | 0.979 | 0.924-0.998 | <0.0001 | 0.971 | 0.900-0.996 | <0.0001 |  |
| *Id* | 0.64 | 92 | 100 | 0.989 | 0.945-1.000 | 100 | 74 | <0.0001 | 0.987 | 0.937-1.000 | <0.0001 | 0.982 | 0.917-0.999 | <0.0001 |  |
| *Idm* | 0.62 | 92 | 100 | 0.989 | 0.945-1.000 | 100 | 74 | <0.0001 | 0.987 | 0.937-1.000 | <0.0001 | 0.982 | 0.917-0.999 | <0.0001 |  |
| *Idmn* | 0.98 | 71 | 100 | 0.824 | 0.737-0.892 | 100 | 45 | <0.0001 | 0.811 | 0.715-0.886 | <0.0001 | 0.803 | 0-691-0.888 | <0.0001 |  |
| *Idn* | 0.91 | 82 | 100 | 0.917 | 0.846-0.962 | 100 | 57 | <0.0001 | 0.904 | 0.823-0.956 | <0.0001 | 0.903 | 0.808-0.961 | <0.0001 |  |
| *Imc1* | -0.05 | 93 | 90 | 0.941 | 0.876-0.978 | 98 | 75 | <0.0001 | 0.934 | 0.861-0.975 | <0.0001 | 0.930 | 0.843-0.977 | <0.0001 |  |
| *Imc2* | 0.40 | 76 | 90 | 0.837 | 0.751-0.902 | 97 | 47 | <0.0001 | 0.830 | 0.736-0.901 | <0.0001 | 0.831 | 0.722-0.910 | <0.0001 |  |
| *InverseVariance* | 0.48 | 86 | 100 | 0.917 | 0.846-0.962 | 100 | 63 | <0.0001 | 0.901 | 0.820-0.954 | <0.0001 | 0.864 | 0.761-0.934 | <0.0001 |  |
| *JointAverage* | 4.36 | 60 | 85 | 0.673 | 0.574-0.763 | 94 | 34 | 0.0024 | 0.664 | 0.557-0.760 | 0.0061 | 0.665 | 0.542-0.773 | 0.0146 |  |
| *JointEnergy* | 0.10 | 87 | 100 | 0.977 | 0.925-0.996 | 100 | 65 | <0.0001 | 0.972 | 0.9.14-0.995 | <0.0001 | 0.962 | 0.887-0.993 | <0.0001 |  |
| *JointEntropy* | 3.72 | 86 | 100 | 0.978 | 0.927-0.997 | 100 | 63 | <0.0001 | 0.974 | 0.916-0.996 | <0.0001 | 0.964 | 0.890-0.994 | <0.0001 |  |
| *MaximumProbability* | 0.18 | 88 | 100 | 0.956 | 0.897-0.987 | 100 | 67 | <0.0001 | 0.948 | 0.880-0.984 | <0.0001 | 0.927 | 0.839-0.975 | <0.0001 |  |
| *MCC* | 0.31 | 82 | 85 | 0.857 | 0.775-0.918 | 96 | 53 | <0.0001 | 0.849 | 0.758-0.916 | <0.0001 | 0.842 | 0.735-0.918 | <0.0001 |  |
| *SumAverage* | 8.72 | 60 | 85 | 0.673 | 0.574-0.763 | 94 | 34 | 0.0024 | 0.664 | 0.557-0.760 | 0.0061 | 0.665 | 0.542-0.773 | 0.0146 |  |
| *SumEntropy* | 2.74 | 81 | 90 | 0.896 | 0.821-0.948 | 97 | 53 | <0.0001 | 0.879 | 0.794-0.938 | <0.0001 | 0.869 | 0.767-0.938 | <0.0001 |  |
| *SumSquares* | 1.02 | 86 | 95 | 0.934 | 0.867-0.973 | 99 | 61 | <0.0001 | 0.922 | 0.846-0.968 | <0.0001 | 0.913 | 0.821-0.967 | <0.0001 |  |
|  |  |  |  |  |  |  |  |  |  |  |  |  |  |  |  |
| ***GLDM*** |  |  |  |  |  |  |  |  |  |  |  |  |  |  |  |
| *GLDM Overall* | 0.48 | 96 | 100 | 0.988 | 0.943-0.999 | 100 | 87 | <0.0001 | 0.986 | 0.934-0.999 | <0.0001 | 0.980 | 0.914-0.999 | <0.0001 | 0.6783 |
| *DependenceEntropy* | 5.84 | 46 | 95 | 0.558 | 0.457-0.656 | 97 | 30 | 0.2817 | 0.549 | 0.440-0.654 | 0.4016 | 0.576 | 0.452-0.693 | 0.2532 |  |
| *DependenceNonUniformity* | 381.84 | 27 | 100 | 0.514 | 0.413-0.614 | 100 | 25 | 0.8077 | 0.507 | 0.400-0.614 | 0.9054 | 0.525 | 0.402-0.646 | 0.7102 |  |
| *DependenceNonUniformityNormalized* | 0.07 | 89 | 100 | 0.958 | 0.899-0.988 | 100 | 69 | <0.0001 | 0.950 | 0.883-0.985 | <0.0001 | 0.949 | 0.868-0.987 | <0.0001 |  |
| *DependenceVariance* | 13.69 | 92 | 100 | 0.967 | 0.912-0.992 | 100 | 74 | <0.0001 | 0.961 | 0.898-0.991 | <0.0001 | 0.963 | 0.888-0.994 | <0.0001 |  |
| *GrayLevelNonUniformity* | 1122.32 | 55 | 95 | 0.713 | 0.616-0.798 | 98 | 34 | <0.0001 | 0.691 | 0.585-0.784 | 0.0004 | 0.678 | 0.556-0.785 | 0.0040 |  |
| *GrayLevelVariance* | 0.99 | 83 | 95 | 0.925 | 0.856-0.968 | 99 | 58 | <0.0001 | 0.912 | 0.834-0.962 | <0.0001 | 0.903 | 0.808-0.961 | <0.0001 |  |
| *HighGrayLevelEmphasis* | 20.61 | 64 | 85 | 0.691 | 0.592-0.778 | 95 | 36 | 0.0006 | 0.682 | 0.576-0.776 | 0.0019 | 0.679 | 0.557-0.786 | 0.0068 |  |
| *LargeDependenceEmphasis* | 73.53 | 88 | 100 | 0.979 | 0.929-0.997 | 99 | 66 | <0.0001 | 0.975 | 0.918-0.996 | <0.0001 | 0.969 | 0.897-0.996 | <0.0001 |  |
| *LargeDependenceHighGrayLevelEmphasis* | 1737.39 | 66 | 95 | 0.755 | 0.660-0.834 | 98 | 40 | <0.0001 | 0.737 | 0.634-0.824 | <0.0001 | 0.743 | 0.625-0.840 | <0.0001 |  |
| *LargeDependenceLowGrayLevelEmphasis* | 3.85 | 95 | 85 | 0.926 | 0.857-0.968 | 96 | 81 | <0.0001 | 0.919 | 0.842-0.966 | <0.0001 | 0.903 | 0.808-0.961 | <0.0001 |  |
| *LowGrayLevelEmphasis* | 0.07 | 55 | 80 | 0.628 | 0.528-0.722 | 92 | 30 | 0.0331 | 0.624 | 0.515-0.724 | 0.0483 | 0.621 | 0.497-0.734 | 0.0835 |  |
| *SmallDependenceEmphasis* | 0.06 | 92 | 100 | 0.970 | 0.916-0.994 | 100 | 74 | <0.0001 | 0.964 | 0.902-0.992 | <0.0001 | 0.950 | 0.870-0.988 | <0.0001 |  |
| *SmallDependenceHighGrayLevelEmphasis* | 1.39 | 92 | 90 | 0.943 | 0.879-0.979 | 97 | 72 | <0.0001 | 0.934 | 0.862-0.976 | <0.0001 | 0.917 | 0.826-0.970 | <0.0001 |  |
| *SmallDependenceLowGrayLevelEmphasis* | 0.01 | 58 | 100 | 0.728 | 0.632-0.811 | 100 | 36 | <0.0001 | 0.713 | 0.608-0.803 | 0.0001 | 0.718 | 0.598.0.819 | 0.0003 |  |
|  |  |  |  |  |  |  |  |  |  |  |  |  |  |  |  |
| ***GLRLM*** |  |  |  |  |  |  |  |  |  |  |  |  |  |  |  |
| *GLRLM Overall* | 0.51 | 92 | 100 | 0.976 | 0.925-0.996 | 100 | 74 | <0.0001 | 0.971 | 0.913-0.995 | <0.0001 | 0.960 | 0.884-0.992 | <0.0001 | 0.5659 |
| *GrayLevelNonUniformity* | 849.26 | 42 | 100 | 0.644 | 0.544-0.736 | 100 | 29 | 0.0074 | 0.622 | 0.514-0.722 | 0.0343 | 0.629 | 0.505-0.742 | 0.0460 |  |
| *GrayLevelNonUniformityNormalized* | 0.26 | 84 | 95 | 0.927 | 0.859-0.969 | 99 | 59 | <0.0001 | 0.915 | 0.837-0.963 | <0.0001 | 0.904 | 0.810-0.961 | <0.0001 |  |
| *GrayLevelVariance* | 1.26 | 86 | 90 | 0.914 | 0.842-0.960 | 97 | 60 | <0.0001 | 0.900 | 0.819-0.953 | <0.0001 | 0.887 | 0.789-0.950 | <0.0001 |  |
| *HighGrayLevelRunEmphasis* | 20.66 | 65 | 85 | 0.695 | 0.597-0.782 | 95 | 37 | 0.0005 | 0.687 | 0.581-0.781 | 0.0014 | 0.682 | 0.560-0.788 | 0.0059 |  |
| *LongRunEmphasis* | 2.35 | 94 | 100 | 0.984 | 0.936-0.999 | 100 | 80 | <0.0001 | 0.981 | 0.927-0.998 | <0.0001 | 0.973 | 0.903-0.997 | <0.0001 |  |
| *LongRunHighGrayLevelEmphasis* | 61.06 | 60 | 95 | 0.708 | 0.610-0.793 | 96 | 35 | <0.0001 | 0.699 | 0.594-0.791 | 0.0002 | 0.719 | 0.599-0.820 | 0.0003 |  |
| *LongRunLowGrayLevelEmphasis* | 0.14 | 93 | 85 | 0.902 | 0.828-0.952 | 96 | 74 | <0.0001 | 0.894 | 0.811-0.949 | <0.0001 | 0.873 | 0.772-0.941 | <0.0001 |  |
| *LowGrayLevelRunEmphasis* | 0.07 | 54 | 80 | 0.634 | 0.534-0.727 | 92 | 30 | 0.0262 | 0.630 | 0.522-0.729 | 0.0383 | 0.620 | 0.496-0.733 | 0.0861 |  |
| *RunEntropy* | 3.38 | 52 | 100 | 0.608 | 0.507-0.703 | 100 | 33 | 0.0403 | 0.596 | 0.487-0.698 | 0.0925 | 0.626 | 0.502-0.739 | 0.0519 |  |
| *RunLengthNonUniformity* | 592.61 | 35 | 90 | 0.530 | 0.429-0.629 | 94 | 25 | 0.6140 | 0.546 | 0.438-0.652 | 0.4472 | 0.518 | 0.395-0.639 | 0.7917 |  |
| *RunLengthNonUniformityNormalized* | 0.59 | 87 | 100 | 0.972 | 0.919-0.995 | 100 | 65 | <0.0001 | 0.967 | 0.906-0.993 | <0.0001 | 0.957 | 0.880-0.991 | <0.0001 |  |
| *RunPercentage* | 0.73 | 88 | 100 | 0.975 | 0.924-0.996 | 100 | 67 | <0.0001 | 0.971 | 0.912-0.995 | <0.0001 | 0.962 | 0.887-0.993 | <0.0001 |  |
| *RunVariance* | 0.51 | 94 | 100 | 0.985 | 0.938-0.999 | 100 | 80 | <0.0001 | 0.982 | 0.929-0.999 | <0.0001 | 0.975 | 0.906-0.998 | <0.0001 |  |
| *ShortRunEmphasis* | 0.79 | 93 | 100 | 0.982 | 0.934-0.998 | 100 | 77 | <0.0001 | 0.979 | 0.923-0.998 | <0.0001 | 0.970 | 0.989-0.996 | <0.0001 |  |
| *ShortRunHighGrayLevelEmphasis* | 17.05 | 78 | 85 | 0.807 | 0.717-0.878 | 96 | 49 | <0.0001 | 0.800 | 0.702-0.877 | <0.0001 | 0.791 | 0.677-0.879 | <0.0001 |  |
| *ShortRunLowGrayLevelEmphasis* | 0.06 | 48 | 80 | 0.530 | 0.429-0.629 | 91 | 27 | 0.6206 | 0.526 | 0.418-0.632 | 0.6805 | 0.519 | 0.396-0.640 | 0.7851 |  |
|  |  |  |  |  |  |  |  |  |  |  |  |  |  |  |  |
| ***GLSZM*** |  |  |  |  |  |  |  |  |  |  |  |  |  |  |  |
| *GLSZM Overall* | 0.53 | 95 | 100 | 0.984 | 0.936-0.999 | 100 | 83 | <0.0001 | 0.981 | 0.927-0.998 | <0.0001 | 0.973 | 0.903-0.997 | <0.0001 | 0.6399 |
| *GrayLevelNonUniformity* | 16.43 | 39 | 90 | 0.560 | 0.459-0.658 | 94 | 26 | 0.3165 | 0.575 | 0.466-0.679 | 0.2315 | 0.550 | 0.426-0.669 | 0.4686 |  |
| *GrayLevelNonUniformityNormalized* | 0.22 | 57 | 95 | 0.782 | 0.690-0.857 | 98 | 35 | <0.0001 | 0.770 | 0.669-0.852 | <0.0001 | 0.769 | 0.653-0.861 | <0.0001 |  |
| *GrayLevelVariance* | 3.56 | 80 | 95 | 0.888 | 0.811-0.942 | 99 | 53 | <0.0001 | 0.976 | 0.789-0.936 | <0.0001 | 0.844 | 0.738-0.920 | <0.0001 |  |
| *HighGrayLevelZoneEmphasis* | 21.42 | 61 | 85 | 0.724 | 0.627-0.808 | 94 | 35 | <0.0001 | 0.714 | 0.609-0.804 | 0.0002 | 0.715 | 0.595-0.817 | 0.0007 |  |
| *LargeAreaEmphasis* | 18401.35 | 64 | 100 | 0.808 | 0.718-0.879 | 100 | 40 | <0.0001 | 0.784 | 0.684-0.863 | <0.0001 | 0.762 | 0.645-0.856 | <0.0001 |  |
| *LargeAreaHighGrayLevelEmphasis* | 433128.83 | 58 | 100 | 0.737 | 0.641-0.819 | 100 | 36 | <0.0001 | 0.711 | 0.606-0.802 | <0.0001 | 0.705 | 0.584-0.808 | 0.0007 |  |
| *LargeAreaLowGrayLevelEmphasis* | 839.89 | 76 | 95 | 0.861 | 0.779-0.921 | 98 | 49 | <0.0001 | 0.844 | 0.752-0.912 | <0.0001 | 0.820 | 0.710-0.902 | <0.0001 |  |
| *LowGrayLevelZoneEmphasis* | 0.14 | 48 | 80 | 0.591 | 0.490-0.687 | 91 | 27 | 0.1555 | 0.586 | 0.477-0.689 | 0.1952 | 0.573 | 0.449-0.691 | 0.3110 |  |
| *SizeZoneNonUniformity* | 25.48 | 59 | 90 | 0.698 | 0.599-0.784 | 95 | 27 | 0.0001 | 0.707 | 0.602-0.798 | 0.0001 | 0.679 | 0.557-0.786 | 0.0044 |  |
| *SizeZoneNonUniformityNormalized* | 0.27 | 77 | 80 | 0.789 | 0.698-0.863 | 91 | 42 | <0.0001 | 0.814 | 0.718-0.888 | <0.0001 | 0.771 | 0.655-0.863 | <0.0001 |  |
| *SmallAreaEmphasis* | 0.53 | 78 | 80 | 0.807 | 0.718-0.878 | 94 | 47 | <0.0001 | 0.831 | 0.737-0.902 | <0.0001 | 0.790 | 0.676-0.878 | <0.0001 |  |
| *SmallAreaHighGrayLevelEmphasis* | 12.97 | 77 | 75 | 0.796 | 0.705-0.869 | 93 | 44 | <0.0001 | 0.799 | 0.701-0.876 | <0.0001 | 0.786 | 0.672-0.875 | <0.0001 |  |
| *SmallAreaLowGrayLevelEmphasis* | 0.05 | 33 | 90 | 0.513 | 0.413-0.613 | 93 | 24 | 0.8288 | 0.521 | 0.413-0.627 | 0.7451 | 0.508 | 0.386-0.630 | 0.9101 |  |
| *ZoneEntropy* | 4.61 | 48 | 85 | 0.592 | 0.491-0.688 | 93 | 28 | 0.1118 | 0.583 | 0.474-0.686 | 0.1716 | 0.569 | 0.445-0.687 | 0.3088 |  |
| *ZonePercentage* | 0.05 | 86 | 100 | 0.946 | 0.884-0.981 | 100 | 63 | <0.0001 | 0.936 | 0.865-0.977 | <0.0001 | 0.917 | 0.826-0.970 | <0.0001 |  |
| *ZoneVariance* | 18072.36 | 64 | 100 | 0.802 | 0.712-0.874 | 100 | 40 | <0.0001 | 0.780 | 0.680-0.861 | <0.0001 | 0.759 | 0.642-0.853 | <0.0001 |  |
|  |  |  |  |  |  |  |  |  |  |  |  |  |  |  |  |
| ***NGTDM*** |  |  |  |  |  |  |  |  |  |  |  |  |  |  |  |
| *NGDTM Overall* | 0.65 | 88 | 100 | 0.966 | 0.911-0.992 | 100 | 67 | <0.0001 | 0.960 | 0.896-0.990 | <0.0001 | 0.957 | 0.880-0.991 | <0.0001 | 0.7348 |
| *Busyness* | 7.30 | 69 | 70 | 0.695 | 0.596-0.782 | 91 | 35 | 0.0006 | 0.665 | 0.558-0.761 | 0.0076 | 0.649 | 0.526-0.759 | 0.0258 |  |
| *Coarseness* | 0.002 | 40 | 100 | 0.596 | 0.495-0.692 | 100 | 29 | 0.0758 | 0.569 | 0.461-0.673 | 0.2351 | 0.593 | 0.469-0.709 | 0.1568 |  |
| *Complexity* | 18.12 | 82 | 95 | 0.830 | 0.743-0.897 | 99 | 56 | <0.0001 | 0.840 | 0.748-0.909 | <0.0001 | 0.824 | 0.714-0.905 | <0.0001 |  |
| *Contrast* | 0.02 | 84 | 95 | 0.901 | 0.827-0.951 | 99 | 59 | <0.0001 | 0.890 | 0.806-0.946 | <0.0001 | 0.886 | 0.788-0.950 | <0.0001 |  |
| *Strength* | 0.06 | 60 | 75 | 0.655 | 0.555-0.746 | 91 | 31 | 0.0077 | 0.626 | 0.518-0.726 | 0.0414 | 0.609 | 0.485-0.723 | 0.1060 |  |
|  |  |  |  |  |  |  |  |  |  |  |  |  |  |  |  |
| ***Shape*** |  |  |  |  |  |  |  |  |  |  |  |  |  |  |  |
| *Shape Overall* | 0.72 | 78 | 95 | 0.927 | 0.858-0.969 | 99 | 51 | <0.0001 | 0.926 | 0.852-0.971 | <0.0001 | 0.915 | 0.824-0.968 | <0.0001 | 0.7807 |
| *Elongation* | 0.80 | 42 | 100 | 0.667 | 0.568-0.757 | 100 | 29 | 0.0029 | 0.676 | 0.570-0.771 | 0.0023 | 0.677 | 0.555-0.784 | 0.0055 |  |
| *Flatness* | 0.47 | 75 | 75 | 0.777 | 0.684-0.853 | 93 | 42 | <0.0001 | 0.780 | 0.680-0.861 | <0.0001 | 0.778 | 0.663-0.869 | <0.0001 |  |
| *LeastAxisLength* | 10.21 | 75 | 100 | 0.850 | 0.766-0.913 | 100 | 49 | <0.0001 | 0.823 | 0.728-0.895 | <0.0001 | 0.797 | 0.684-0.884 | <0.0001 |  |
| *MajorAxisLength* | 29.17 | 52 | 85 | 0.699 | 0.601-0.785 | 94 | 30 | 0.0007 | 0.674 | 0.567-0.769 | 0.0051 | 0.664 | 0.541-0.773 | 0.0142 |  |
| *Maximum2DDiameterColumn* | 27.37 | 61 | 95 | 0.772 | 0.679-0.849 | 98 | 37 | <0.0001 | 0.751 | 0.648-0.836 | <0.0001 | 0.744 | 0.626-0.841 | <0.0001 |  |
| *Maximum2DDiameterRow* | 27.49 | 55 | 95 | 0.770 | 0.677-0.848 | 98 | 34 | <0.0001 | 0.749 | 0.646-0.834 | <0.0001 | 0.728 | 0.608-0.828 | 0.0001 |  |
| *Maximum2DDiameterSlice* | 32.78 | 46 | 85 | 0.652 | 0.552-0.743 | 93 | 27 | 0.0126 | 0.624 | 0.515-0.724 | <0.0001 | 0.615 | 0.491-0.729 | 0.0946 |  |
| *Maximum3DDiameter* | 37.67 | 46 | 95 | 0.710 | 0.612-0.795 | 98 | 30 | 0.0001 | 0.681 | 0.574-0.775 | 0.0025 | 0.679 | 0.557-0.786 | 0.0051 |  |
| *MeshVolume* | 3632.46 | 61 | 100 | 0.801 | 0.711-0.873 | 100 | 39 | <0.0001 | 0.778 | 0.678-0.859 | <0.0001 | 0.752 | 0.634-0.847 | <0.0001 |  |
| *MinorAxisLength* | 17.28 | 65 | 90 | 0.777 | 0.684-0.853 | 96 | 38 | <0.0001 | 0.760 | 0.658-0.844 | <0.0001 | 0.742 | 0.623-0.839 | <0.0001 |  |
| *Sphericity* | 0.68 | 57 | 80 | 0.617 | 0.516-0.712 | 92 | 31 | 0.0426 | 0.631 | 0.522-0.730 | 0.0314 | 0.624 | 0.500-0.737 | 0.0661 |  |
| *SurfaceArea* | 1629.97 | 64 | 95 | 0.772 | 0.679-0.849 | 98 | 39 | <0.0001 | 0.746 | 0.644-0.832 | <0.0001 | 0.723 | 0.603-0.823 | 0.0002 |  |
| *SurfaceVolumeRatio* | 0.49 | 69 | 100 | 0.820 | 0.733-0.889 | 100 | 44 | <0.0001 | 0.791 | 0.692-0.869 | <0.0001 | 0.779 | 0.664-0.869 | <0.0001 |  |
| *VoxelVolume* | 3657.27 | 63 | 100 | 0.803 | 0.713-0.875 | 100 | 39 | <0.0001 | 0.779 | 0.680-0.860 | <0.0001 | 0.753 | 0.635-0.848 | <0.0001 |  |
|  |  |  |  |  |  |  |  |  |  |  |  |  |  |  |  |
|  |  |  |  |  |  |  |  |  |  |  |  |  |  |  |  |
| ***2) CT iodine uptake*** |  |  |  |  |  |  |  |  |  |  |  |  |  |  |  |
| *Mean attenuation (HU)* | 35.90 | 70 | 65 | 0.637 | 0.536-0.730 | 89 | 34 | 0.0407 | 0.679 | 0.571-0.774 | 0.0104 | 0.691 | 0.569-0.797 | 0.0089 | 0.5861 |
| *Iodine uptake (mg/mL)* | 1.40 | 89 | 70 | 0.852 | 0.767-0.914 | 92 | 58 | <0.0001 | 0.838 | 0.745-0.908 | <0.0001 | 0.835 | 0.726-0.914 | <0.0001 | 0.8297 |
| *Fat fraction (%)* | 15.10 | 61 | 80 | 0.636 | 0.535-0.729 | 93 | 33 | 0.0305 | 0.688 | 0.591-0.794 | 0.0064 | 0.707 | 0.585-0.811 | 0.0027 | 0.4463 |
|  |  |  |  |  |  |  |  |  |  |  |  |  |  |  |  |
| ***3) MRI ADC Mapping*** |  |  |  |  |  |  |  |  |  |  |  |  |  |  |  |
| *ADC value (mm^2^/s)* | 1.239 | 51 | 85 | 0.690 | 0.587-0.780 | 93 | 32 | 0.0008 | 0.725 | 0.616-0.817 | 0.0001 | 0.769 | 0.649-0.869 | <0.0001 | 0.3075 |
|  |  |  |  |  |  |  |  |  |  |  |  |  |  |  |  |
| ***4) Overall radiomics, CT iodine uptake, and MRI ADC Mapping*** | 0.03 | 98 | 100 | 0.996 | 0.894-1.000 | 100 | 96 | <0.0001 | 0.995 | 0.884-1.000 | <0.0001 | 0.993 | 0.846-1.000 | <0.0001 | 0.9089 |
|  |  |  |  |  |  |  |  |  |  |  |  |  |  |  |  |
|  |  |  |  |  |  |  |  |  |  |  |  |  |  |  |  |
|  |  |  |  |  |  |  |  |  |  |  |  |  |  |  |  |

*Abbreviations: AUC, area under the curve. PPV, positive predictive value. NPV, negative predictive value. GLCM, Gray-Level Co-Occurrence Matrix. GLDM, Gray-Level Dependence Matrix. GLRLM, Grey-Level Run Length Matrix. GLSZM, Gray-Level Size Zone Matrix. NGTDM, Neighboring Gray Tone Difference Matrix. HU, Hounsfield unit. RU, relative unit. ADC, apparent diffusion coefficient.*
