# Supplementary material for: Multiparametric detection and outcome prediction of pancreatic cancer involving dual-energy CT, diffusion-weighted MRI, and radiomics
Source: Cancer Imaging. 2023 Apr 18;23:38. doi: 10.1186/s40644-023-00549-8 (PMC10114410; doi:10.1186/s40644-023-00549-8)
Supplement: Supplementary file 3 — Additional file 3:Supplemental Table 3. Comparison of ROC curves malignant/normal vs. malignant/inflamed. [file 40644_2023_549_MOESM3_ESM.docx]

**Supplemental Table 3:** Comparison of ROC Curves Malignant/Normal vs. Malignant/Inflamed (significant values are written in bold).

| **Variables** | **AUC overall**  p-value (t-test malignant/normal vs. malignant/inflamed) | **AUC train**  p-value (t-test malignant/normal vs. malignant/inflamed) | **AUC test**  p-value (t-test malignant/normal vs. malignant/inflamed) |
| --- | --- | --- | --- |
|  |  |  |  |
| **1) CT radiomics texture features** |  |  |  |
|  |  |  |  |
| **Radiomics Overall** | 0.3649 | 0.3410 | 0.4108 |
|  |  |  |  |
| **First-order** |  |  |  |
| First-order Overall | 0.1155 | 0.1226 | 0.1159 |
|  |  |  |  |
| **GLCM** |  |  |  |
| GLCM Overall | 0.3121 | 0.3208 | 0.3073 |
|  |  |  |  |
| **GLDM** |  |  |  |
| GLDM Overall | 0.6513 | 0.7061 | 0.6195 |
|  |  |  |  |
| **GLRLM** |  |  |  |
| GLRLM Overall | 0.3761 | 0.3684 | 0.3343 |
|  |  |  |  |
| **GLSZM** |  |  |  |
| GLSZM Overall | 0.4073 | 0.4497 | 0.4110 |
|  |  |  |  |
| **NGTDM** |  |  |  |
| NGDTM Overall | 0.4828 | 0.4653 | 0.6775 |
|  |  |  |  |
| **Shape** |  |  |  |
| Shape Overall | 0.4121 | 0.4295 | 0.5350 |
|  |  |  |  |
|  |  |  |  |
| **2) CT iodine uptake** |  |  |  |
| Mean attenuation (HU) | 0.5698 | 0.5041 | 0.5791 |
| Iodine uptake (mg/mL) | **0.0190** | **0.0163** | **0.0245** |
| Fat fraction (%) | 0.4278 | 0.3823 | 0.4727 |
|  |  |  |  |
|  |  |  |  |
| **3) MRI ADC Mapping** |  |  |  |
| ADC value (mm^2^/s) | **0.0106** | 0.0819 | 0.3810 |
|  |  |  |  |
|  |  |  |  |
| **4) Overall radiomics, CT iodine uptake, and MRI ADC Mapping** | **0.0127** | 0.1102 | 0.2684 |
|  |  |  |  |
|  |  |  |  |

*Abbreviations: AUC, area under the curve. GLCM, Gray-Level Co-Occurrence Matrix. GLDM, Gray-Level Dependence Matrix. GLRLM, Grey-Level Run Length Matrix. GLSZM, Gray-Level Size Zone Matrix. NGTDM, Neighboring Gray Tone Difference Matrix. HU, Hounsfield unit. RU, relative unit. ADC, apparent diffusion coefficient.*
